# Partners in crime: Tbf1 and Vid22 promote expansions of long human telomeric repeats at an interstitial chromosome position in yeast

**DOI:** 10.1093/pnasnexus/pgac080

**Published:** 2022-06-08

**Authors:** Elina A Radchenko, Anna Y Aksenova, Kirill V Volkov, Alexander A Shishkin, Youri I Pavlov, Sergei M Mirkin

**Affiliations:** Department of Biology, Tufts University, Medford, MA 02421, USA; Department of Biology, Tufts University, Medford, MA 02421, USA; Laboratory of Amyloid Biology, St. Petersburg State University, St. Petersburg, 199034, Russia; Laboratory of Amyloid Biology, St. Petersburg State University, St. Petersburg, 199034, Russia; The Broad Institute of MIT and Harvard, Cambridge, MA 02139, USA; Eppley Institute for Research In Cancer and Allied Diseases, Omaha, NE 68198, USA; Department of Biology, Tufts University, Medford, MA 02421, USA

**Keywords:** interstitial telomeric sequences, Tbf1 protein, Vid22 protein, repeat expansion, DNA replication

## Abstract

In humans, telomeric repeats (TTAGGG)_n_ are known to be present at internal chromosomal sites. These interstitial telomeric sequences (ITSs) are an important source of genomic instability, including repeat length polymorphism, but the molecular mechanisms responsible for this instability remain to be understood. Here, we studied the mechanisms responsible for expansions of human telomeric (Htel) repeats that were artificially inserted inside a yeast chromosome. We found that Htel repeats in an interstitial chromosome position are prone to expansions. The propensity of Htel repeats to expand depends on the presence of a complex of two yeast proteins: Tbf1 and Vid22. These two proteins are physically bound to an interstitial Htel repeat, and together they slow replication fork progression through it. We propose that slow progression of the replication fork through the protein complex formed by the Tbf1 and Vid22 partners at the Htel repeat cause DNA strand slippage, ultimately resulting in repeat expansions.

Significance Statement Telomeric repeats that are present at internal chromosomal sites lead to genome instability in health and disease. We studied the mechanisms of instability of human interstitial telomeric repeats in a yeast experimental system. We found that it is mediated by the complex of two proteins, Tbf1 and Vid22, bound to those repeats. Since both proteins have human counterparts, our discovery points to the targets potentially responsible for the instability of interstitial telomeric repeats in human cells.

## Introduction

Telomeres, special nucleoprotein structures at the ends of chromosomes, play a key role in genome stability by protecting chromosomal ends and tackling the end-replication problem (1–3). Formation of a protective cap at the ends of chromosomes in mammals depends on several protein factors interacting with simple telomeric repeats (TTAGGG)_n_. The six-subunit protein complex shelterin is essential for telomere maintenance in vertebrates (4). It consists of TRF1, TRF2, POT1, TPP1, TIN2, and RAP1 proteins. A total of three of these proteins, TRF1, TRF2, and POT1, directly interact with telomeric DNA: TRF1 and TRF2 interact with double-stranded telomeric repeat, while POT1 interacts with a single-stranded G-rich overhang at the tip of the telomere.

Lower eukaryotes, such as budding yeast, lack the shelterin complex; instead, the Rap1 protein (an ortholog of human RAP1) binds to double-stranded telomeric repeats. Yeast telomeres are typically short and carry tracts of irregular sequence (TG_1-3_)_n_, whereas vertebrate telomeres are much longer and composed of nearly perfect (TTAGGG)_n_ repeats.

Structural alterations of telomeric DNA were also implicated in chromosome stability and maintenance. A substantial body of evidence demonstrates that capping of telomeric ends is achieved via the formation of T-loops. These are the structures formed upon invasion of single-stranded telomeric 3’-ends into adjacent duplex DNA that are additionally stabilized by the presence of the TRF2 (and possibly other) proteins (5, 6). Besides T-loops, single-stranded telomeric ends can form G4-DNA: a four-stranded DNA structure built of stacked G-quadruplexes (7, 8). The role of G4-DNA in the chromosome end-protection and telomere maintenance is also broadly discussed. Importantly both T-loops and G4-DNA are formed by human telomeric (Htel) repeats in vivo (6, 9).

Telomeric repeats are also found at various loci inside the genome in many organisms and their function at these loci is not fully understood. Such sequences are called Interstitial Telomeric Sequences (ITSs) and they can significantly vary in size and quantity in different genomes. The human genome carries short ITSs (s-ITSs) tracts in multiple locations and a few long, fusion ITSs which originate from telomere–telomere fusion of ancestral chromosomes (10–12). Little is known about the functional role of such sequences, although recent data relate ITSs with the maintenance of telomeric loops, genome stability, and regulation of gene expression (13–16). ITSs often colocalize with sites of fragility, hyperrecombination, and chromosomal aberrations and coincide with chromosomal translocations and abnormalities found in various human diseases (17–31). Like other microsatellites, s-ITSs show substantial length polymorphism (12, 30, 32–34). Importantly, the length of the s-ITSs tract becomes destabilized in some tumors (30, 32). There is mounting evidence that shelterin components occupy selective interstitial telomeric sites in human genome. TRF1, TRF2, RAP1, and TIN2 were found at ITS regions outside of telomeres, where they are thought to impact stability of these sequences and regulate transcription (11, 29, 35–38).

Interestingly, budding yeast also carry a few copies of human TTAGGG repeats within subtelomeric Y’ and X regions (39–41). These elements are called STAR-repeats (subtelomeric antisilencing regions) since they function as insulators by blocking heterochromatin from spreading from telomeric regions inside chromosome. STAR-repeats are bound by the Tbf1 (TTAGGG binding factor 1) protein, a homolog of human TRF1 and TRF2 proteins (39, 42–44).

Using a yeast model system, we have previously shown that short tracts of perfect yeast telomeric (Ytel) repeats, when artificially placed inside a chromosome, are able to induce gross chromosomal rearrangements and mutagenesis in adjacent loci (45). They also undergo spontaneous tract length changes by either expanding or contracting (45, 46). This instability results from the formation of double-stranded breaks or single-stranded gaps within Ytel-repeats during their replication and subsequent repair of these lesions via homologous DNA recombination or postreplication DNA repair (46).

Here, we examined the stability of Htel repeats placed into the body of yeast chromosome III. Similar to Ytel repeats, they appear to undergo expansions with high frequency. Despite this similarity, the genetic control of Htel repeat instability differs dramatically from that of Ytel repeats. First, it depends on the functionality of the Tbf1 protein, as a deletion within its N-terminal insulation domain (*tbf1Δi*) dramatically (more than 100-fold) stabilizes the repeat. Second, it also depends on the presence of the Tbf1 partner, Vid22: deletion of the *VID22* gene stabilizes the repeat to the same extent as the mutant *tbf1Δi* allele. Using 2D electrophoretic analysis of replication intermediates, we found that replication fork stalls at the Htel repeat, and this stalling is decreased in both the *tbf1Δi* and *vid22Δ* mutants. Finally, the rate of Htel repeat instability is decreased in yeast strains carrying mutant alleles of replicative DNA polymerases, which is particularly pronounced in DNA polymerase epsilon mutants. We hypothesize that binding of the Tbf1 and Vid22 proteins to the Htel repeat results in the slow replication through the repeat provokes strand slippage, which ultimately leads to its expansion.

## Materials and Methods

### Plasmids

Plasmids with Htel repeats in the *URA3* reporter cassette were constructed during this study. Construction of these and other plasmids is described in the Supplementary Material.

### Strains

Strains are listed in Table S1 (Supplementary Material), individual gene deletions were obtained via PCR-based method for gene replacement (47, 48). PCR cassettes for gene replacements were obtained using plasmids pAG32 (*hphMX4* cassette), pRS303 (*HIS3* cassette), pFA6-kanMX4 (*kanMX4* cassette), pAG25 (*natMX4* cassette), and pUC19-hphMX4 (*hphMX4* cassette). Primers used to create these cassettes are listed in Table S2 (Supplementary Material). Strains carrying the *TBF1* or *tbf1Δi* fused with 13xMyc tag were constructed as follows: genomic DNA from strains YVR032 and YVR118, described in (49) was used for PCR to amplify *TBF1-13xMyc-HIS3MX6* and *tbf1Δi-13xMyc-HIS3MX6* alleles, respectively. Genomic DNA from YVR032 was also used to amplify *VID22-13xMyc-HIS3MX6*. These PCR products were integrated into the SMY758 strain to get SMY904, SMY901, and SMY1075 strains (Table S1, Supplementary Material). Mutations in the *POL2* and *POL3* genes encoding the catalytic subunits of the DNA polymerases epsilon and delta were constructed via pop-in pop-out method as described in (50). All these polymerase mutations were obtained in the ΔI(-2)I-7B-YUNI300 strain background (51). The *UR-(CCCTAA)_38_-A3* cassette was then introduced into these strains. The strain with *tbf1Δi* without 13xMyc tag (SMY1076) used in the chromatin immunoprecipitation (ChIP) experiment was obtained using a simplified CRISPR-Cas9 approach using the pRCC-N plasmid (Addgene) (52).

The *tbf1Δi-13xMyc-HIS3MX6* or *vid22Δ* alleles were integrated into the SMY710 strain, to create SMY1070 and SMY1106 strains, respectively. All these three strains were then transformed with the plasmid pRS425-7-18B that contains the (CCCTAA)_60_ repeat (Figure S1, Supplementary Material). The *wt* SMY710 strain was also transformed with the control pRS425-UIRL plasmid without Htel repeats.

### ChIP

Yeast were grown in 40 mL liquid YPD at 30°C to reach OD_600_ = 1.0. After cross-linking with 1% formaldehyde, cells were lysed using glass beads and ChIP lysis buffer (50 mM HEPES/KOH, 140 mM NaCl, 1 mM EDTA, 1% Triton X-100, and 0.1% Na-deoxycholate) in a Mini-Beadbeater Homogenizer (Biospec products). Then cells were sonicated using Diagenode bioruptur (three cycles of 1 minute sonication and 1 minute pause). Immunoprecipitation (IP) was performed using 1 μL of anti-Myc (9E10 sc-40 Santa-Cruz) antibodies and then protein G beads. After cross-linking was reversed, DNA was purified using phenol–chloroform extraction. ChIP and input samples were analyzed by duplex PCR using two sets of primers: ChrV-fwd and ChrV-rev for the normalization in combination with UIRL1 and UIRL2 followed by gel electrophoresis (Table S2, Supplementary Material). The gels were then scanned using the Amersham Typhoon laser scanner with 532 nm laser and Cy3 filter. Intensity of the bands for IP and input samples was measured by NIH ImageJ software. Then IP/input values for the cassette band were normalized to IP/input values for the ChrV fragment. SEM for three or four independent experiments was calculated and the difference between the strains was evaluated with t test.

### Analysis of replication intermediates by 2D gel electrophoresis

The protocol of yeast plasmid 2D gels was modified from (53–55). A volume of 50 mL of overnight yeast cultures grown in selective Leu^–^ YNB media were diluted with 350 mL of YPD to have OD_600_ = 0.2. When the OD_600_ reached 2.0, cell cultures were poured into 500 mL centrifuge tubes with frozen 0.2 M EDTA and cell growth was stopped by adding 4 mL of 10% NaN3. Cultures were shaken until EDTA was thawed. Cells were pelleted for 5 minutes at 2,500 rpm, washed twice with cold water and frozen at −80°C. The next day, cells were resuspended in 5 mL NIB buffer (17% glycerol, 50 mM morpholinepropanesulfonic acid, 150 mM NaOAc, 2 mM MgCl2, 0.5 mM spermidine, 0.15 mM spermine, and pH 7.2) and 50 μL of zymolyase (1,000 U). After 1 hour of incubation at 30°C, an additional 50 μL of zymolyase was added. After centrifugation at 5,000 rpm for 20 minutes, cells were resuspended in 5 mL of G2 Buffer from the Qiagen Blood and Cell Culture DNA Midi Kit. Lysates were treated with 150 μL of proteinase K for a total of 1.5 to 2 hours. After centrifugation at 9,000 rpm for 10 minutes, the supernatant was mixed with an equal (5 mL) amount of QBT buffer and loaded on a Qiagen Genomic-tip 100/G column. The column was washed twice with 7.5 mL buffer QC. DNA was eluted with 5 mL of QF buffer followed by isopropanol precipitation. The DNA was resuspended in 200 μL of elution buffer or TE.

A volume of 25 μg of DNA was digested with 20 μL of ScaI and 20 μL of AflII for more than 10 hours. Digested DNA was purified with phenol–chloroform, eluted in 22 μL of water and loaded into the gel.

The first dimension was run in 0.4% in 1xTBE agarose gel at 30 V (1 V/cm) for 24 hours, the second dimension was run in 1% 1xTBE agarose gel at 150 V (5 V/cm) for 9 hours in the presence of 0.3 μg/mL ethidium bromide. The gels were incubated for 12 to 15 minutes in 0.25 N HCl and then washed three times with water. Transfer to a charged nylon membrane (Hybond-XL, GE Healthcare) was performed in 0.4 N NaOH. Hybridization of the membrane was performed overnight at 65°C with a 394 bp P^32^ primed probe, corresponding to the Amp^R^ sequence of the pRS425 plasmid. Membranes were washed twice with the washing solution I (SSC 2× and 1% SDS) and twice with the washing solution II (SSC 0.1× and 0.1% SDS) for 15 minutes per wash. Membranes were exposed on IR-sensitive screens for 7 to 10 days and detection was performed on Amersham Typhoon Scanner Platform. Quantifications were performed with the NIH ImageJ program and a custom R-script as described in (53) and in the Supplemental Material.

### Measurements of rates of expansion, contraction, and gene inactivation

Rates were calculated using the Ma-Sandri-Sarkar maximum likelihood estimator (MSS-MLE) method with correction for plating efficiency (VZ-MLE) as described earlier (46, 56). At least 12 independent cultures were studied for each strain. At least 48 colonies grown on the 5-FOA media in the fluctuation test were analyzed by PCR to evaluate the length of the repetitive tract. The distribution of the expanded clones in the analyzed independent cultures was used to calculate the rates of expansion. In total, 95% CI (error bars) were calculated based on distribution of expanded clones in independent cultures using the MSS-MLE with a correction for sampling efficiency (MSS-MLE and VZ-MLE) as described in (46, 56).

### Other methods

Selection of 5-FOA resistant colonies was performed on 0.1% 5-FOA media and analyzed by colony PCR as described in detail previously (46, 56). Sequencing was performed at the University of Chicago Sequencing Facility, at GENEWIZ (www.genewiz.com) and at the Research Resource Center for Molecular and Cell Technologies (Research Park, St. Petersburg State University, Russia).

## Results

### Interstitial Htel sequences are unstable and inactivate reporter gene expression in yeast

**Fig. 1.**
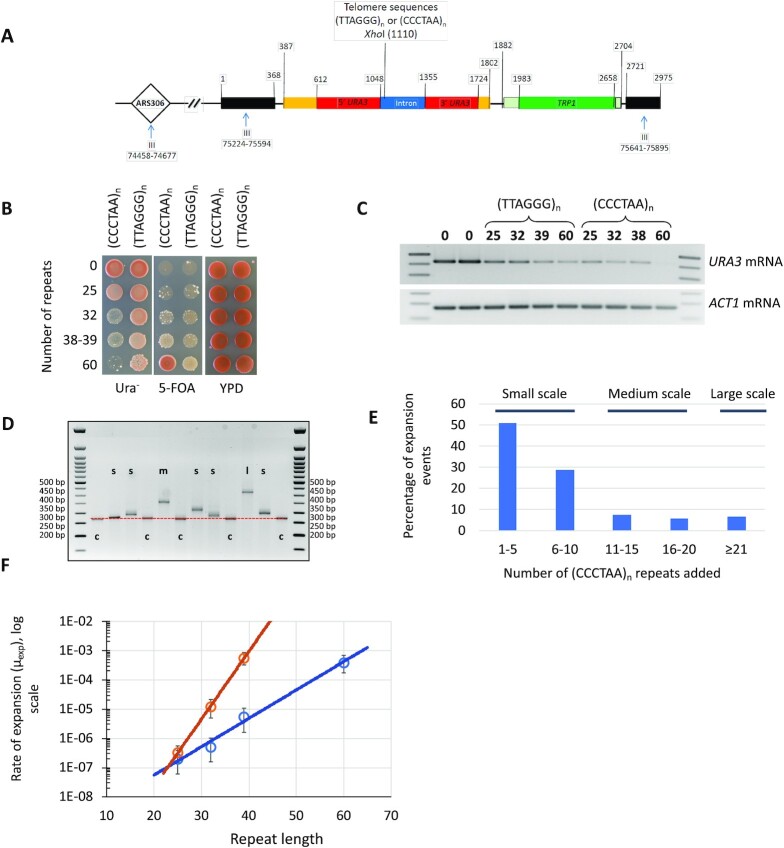
The *URA3-Intron* system used to study instability of Htel repeats. (A) The map of the *URA3-Intron* cassette and its position when integrated in the genome. The cassette contains flanking sequences from chromosome III (black), flanking and coding sequences from the *URA3* gene (yellow and red, respectively), an intron sequence derived from the *ACT1* gene from chromosome VI (blue), and the *TRP1* flanking and coding sequences (pale green and dark green, respectively). Htel repeats were inserted in both orientations into the indicated XhoI site (blunted) within the intron of the *URA3* gene. Numbers above the cassette indicate the position in the cassette, and numbers below the line are the SGD coordinates for S288C reference genome. (B) Phenotypes of strains carrying Htel repeats in the *URA3-Intron* reporter gene. Suspensions containing approximately equal amounts of cells were plated as drops on three different types of medium: 5-FOA-containing synthetic media, synthetic media without uracil, and complete YPD media. (C) Expression of the *URA3-Intron* gene in strains with insertions of Htel repeats. We examined expression of the *URA3-Intron* gene by RT-PCR in strains carrying 25, 32, 39, and 60 copies of Htel repeat in G-orientation and strains carrying 25, 32, and 38 copies of Htel repeat in C-orientation. The strain carrying no repeats in the *URA3-Intron* gene was used as a control. The rows labeled *URA3* and *ACT1* mRNA show the relative amounts of spliced *URA3* mRNA and the control actin mRNA, respectively. (D) Gel electrophoresis of the PCR analysis of 5-FOA^R^ colonies derived from a fluctuation test of a strain with the (CCCTAA)_38_ repeat. c—nonexpanded (CCCTAA)_38_ repeat (270 bp), s—small-scale expansion (1 to 10 repeats added), m—medium-scale expansion (11 to 20 repeats added), and l—large-scale expansion (more than 20 repeats added); red dashed line shows nonexpanded (CCCTAA)_38_ repeat (270 bp PCR product); 2% TBE-agarose gel; 50 bp Quick-load ladder (NEB) is shown on both sides of the gel. (E) Length distribution of expanded repeats among 5-FOA^R^ clones in the *wt* strain carrying the (CCCTAA)_38_ repeat. (F) Htel repeats are prone to expansions in both orientations and the expansion rate correlates with the tract length. Blue with circles trend is the rate of (TTAGGG)_n_ repeat expansions, orange with circles trend is the rate of (CCCTAA)_n_ repeat expansions. Rates were calculated using MSS-MLE and VZ-MLE as described in (46, 56). In total, 95% confidence limits are shown for each spot.

Htel sequences of varying lengths were cloned into an intron of the artificially split *URA3* gene (57). Specifically, we cloned 25, 32, 39, and 60 Htel repeats in G-orientation (TTAGGG)_n_ and 25, 32, 38, and 60 Htel repeats in C-orientation (CCCTAA)_n_ into this split *URA3* cassette (45, 46, 57). The corresponding cassettes carrying the *URA3-Intron* with Htel insertions were inserted close to the *ARS306* on chromosome III (Fig. 1A). The G- or C-orientation of the telomeric tract denotes the sense strand for transcription, which is also the lagging strand template for DNA replication. Similar to Ytel repeats, the *URA3* reporter becomes inactivated as the repeat's length increases (46). The presence of 60 CCCTAA repeats in the *URA3-Intron* makes yeast fully Ura^–^ and resistant to 5-fluoroorotic acid (5-FOA^R^), while the presence of 32-to-38 CCCTAA repeats results in a phenotype, which is partially Ura^+^ and partially 5-FOA^R^. At the same time, a yeast strain with 60 TTAGGG repeats is Ura^+^, bueut is slightly resistant to 5-FOA (Fig.   1B). These phenotypes are due to an obvious decrease in the amount of mature *URA3* mRNA (Fig. 1C): 60 CCCTAA repeats completely eliminate it, while shorter (CCCTAA)_n_ repeats as well as (TTAGGG)_n_ repeats decrease its amount. Thus, inactivation of gene expression by Htel repeat is orientation-dependent, as the gene inactivation is more pronounced in C-orientation of the repeat.

We assumed, therefore, that expansions of shorter Htel repeats would make the corresponding strains Ura^–^ and 5-FOA^R^. To test this assumption, we conducted fluctuation test experiments. In brief, individual colonies of strains carrying Htel repeats of varying lengths that were grown on nonselective (YPD) medium are plated on selective media (5-FOA) followed by PCR analysis of repeat length among the resistant clones (45). Figure 1(D) shows an example of repeat-length analysis in 5-FOA^R^ clones from the strain with the (CCCTAA)_38_ repeat. In accord with our hypothesis, 5-FOA^R^ clones often contain expanded repeats, and one can see expansions ranging from small- to large-scale. Figure 1(E) shows the length distributions of expanded repeats. The majority (80%) of them are small-scale ranging from 1 to 10 repeats added; medium-scale expansions (up to 20 extra repeats) were observed in 13% of 5-FOA^R^ clones, while the remaining 7% corresponded to large-scale (> 21 extra repeats) expansions. This scale distribution is not very surprising given the partial Ura^+^/partial 5-FOA^R^ phenotype of the initial strain with 38 CCCTAA repeats. Note that this system allowed us to study a wide range of expansions at once.

The rates of repeat expansions determined as described in (46, 56) are shown in Fig. 1(F) and Table S3 (Supplementary Material). They are clearly length-dependent in both orientations. Furthermore, the expansion rate seemed significantly higher in the C-orientation of the repeat as compared to the G-orientation (Fig. 1F and Table S3, Supplementary Material). We were concerned, however, that this orientation-dependence could be a selection artifact resulting from a stronger inhibition of the reporter's expression by Htel repeats in the C-orientation (Fig. 1C), which would consequently lower the threshold for repeat expansions in 5-FOA^R^ clones.

To address this concern, we compared the rates for spontaneous tract alterations in individual colonies that grew without any selection between the strains carrying 39 Htel repeats in G-orientation or 38 Htel repeats in C-orientation (Figure S2, Supplementary Material), as described in (45). Since this experiment allowed us to primarily detect small-scale expansions and contractions, comprising vast majority of all instability events, some of which may not be selected for on the 5-FOA media, the observed rates were very high, reaching up to 6 × 10^–3^ per replication. Importantly, however, the rates of repeat tract alteration on the nonselective media did not differ significantly between Htel repeats of the similar length in either orientation, contrary to what was observed under selective pressure. We conclude, therefore, that at least small-scale length instability of Htel repeats is largely orientation independent.

We also sequenced the *URA3* gene and Htel repeats in multiple independent 5-FOA^R^ clones from strains with 25 and 38 to 39 repeats in both orientations. We found that in all cases, expanded repetitive tracts remained uninterrupted. 5-FOA^R^ clones from strains with 38 to 39 repeats did not have genetics changes besides repeat expansions, while 5-FOA^R^ clones from strains with 25 Htel repeats contained point mutations, deletions and rearrangements in the *URA3* gene likely contributing to their 5-FOA resistance (Table S4, Supplementary Material).

### Instability of Htel repeats depends on the presence and/or functionality of protein partners Tbf1 and Vid22

**Fig. 2.**
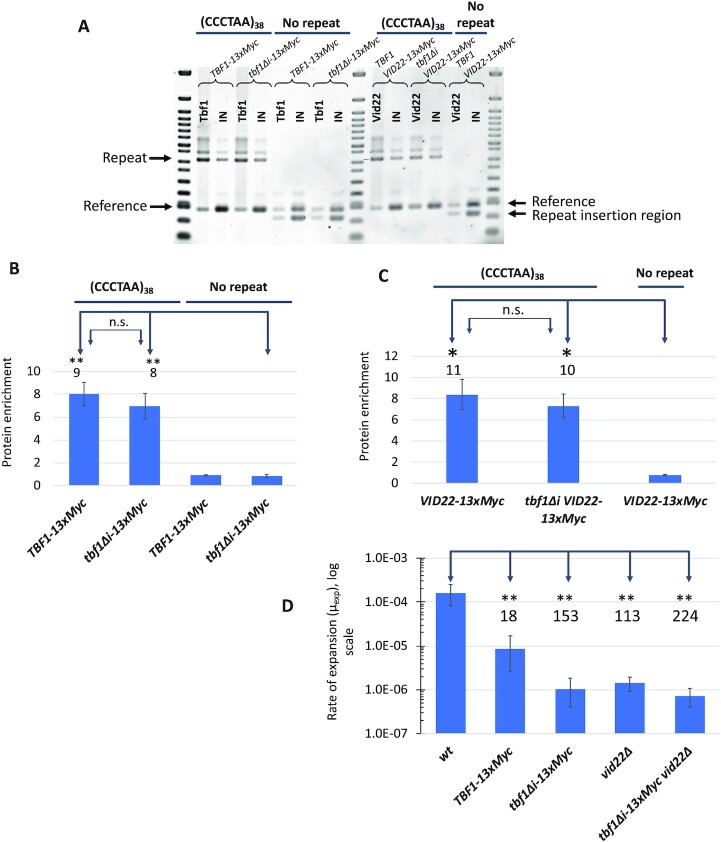
Influence of the *TBF1, tbf1Δi*, and *vid22Δ* on Htel tract stability. (A) ChIP analysis demonstrates that Tbf1-13xMyc, tbf1Δi-13xMyc, and Vid22-13xMyc are enriched at (CCCTAA)_38_ interstitial Htel repeat. ChIP analysis was performed in seven strains: SMY904 (*TBF1-13xMyc*, (CCCTAA)_38_ repeat), SMY901 (*tbf1Δi-13xMyc*, (CCCTAA)_38_ repeat), SMY906 (*TBF1-13xMyc*, no repeats), SMY904 (*tbf1Δi-13xMyc*, no repeats), SMY1075 (*VID22-13xMyc*, (CCCTAA)_38_ repeat), SMY1083 (*tbf1Δi VID22-13xMyc*, (CCCTAA)_38_ repeat), and SMY1081(*VID22-13xMyc*, no repeats) using anti-Myc antibodies that recognize Tbf1, Tbf1Δi, or Vid22 proteins fused with C-terminal 13 × Myc epitope. Duplex PCR analysis of the input (IN) and immunoprecipitated (IP) samples was performed with UIRL1/UIRL2 primers that amplify the repeat (Repeat) and ChrV-fwd/ChrV-rev primers that amplify a chromosome V-specific product (Reference). ChIP method and antibodies are described in Materials and Methods. (B) Densitometry of the ChIP analysis. Optical density of the IP/IN samples for the repeat fragment was normalized to optical density of IP/IN values for the reference fragment. Densitometry methods are described in Materials and Methods. SEM for three or more independent ChIP experiments are shown as error bars. Numbers above the bars indicate protein fold enrichment at the (CCCTAA)_38_ repeat compared to the corresponding strain with no repeats. **—indicate significance at alpha level 0.01. (C) Densitometry of the ChIP analysis. Optical density of the IP/IN samples for the repeat fragment was normalized to optical density of IP/IN values for the reference fragment. Densitometry methods are described in Materials and Methods. SEM for three or more independent ChIP experiments are shown as error bars. Numbers above the bars indicate protein fold enrichment at the (CCCTAA)_38_ repeat compared to the corresponding strain with no repeats. *—indicate significance at alpha level 0.05. (D) Expansion rate of the (CCCTAA)_38_ repeat is decreased in strains carrying *TBF1-13xMyc, tbf1Δi-13xMyc*, and *vid22Δ* alleles. Error bars represent 95% CI. Numbers above the bars indicate fold change in expansion rate in mutant strains compared to the isogenic *wt* (SMY758) strain. **—nonoverlapping 95% CI.

Short Htel (TTAGGG)_n_ repeats are present in the yeast genome in subtelomeric areas, where they are bound by the Tbf1 protein (a homolog of shelterin proteins TRF1 and TRF2). These elements of the yeast genome are called STAR-repeats since they can block heterochromatin from spreading from telomeric regions inside the chromosomes (39, 40, 42–44). It was reasonable to expect, therefore, that Tbf1 will bind interstitial Htel repeats within our cassettes as well. To address this question, we studied binding of the Tbf1 protein with 13xMyc tag added to its C-terminus described in (49) via ChIP analysis. Figures 2(A) and (B) show that Myc-tagged Tbf1 protein is strongly (9-fold) enriched at the Htel tract as compared to the strain with 0 repeats.

*TBF1* is an essential gene. Besides its insulation function at subtelomeric regions, Tbf1 protein is also responsible for transcription of snoRNA and several other genes (39). The protein contains two main domains: SANT/Myb-like, which is responsible for DNA binding, and Insulation domain, which is needed for the insulation function as well as transcription activation (44, 58, 59). A viable mutation called *tbf1Δi* removes a large part of the insulation domain (49, 58, 59). Consequently, the mutant Tbf1Δi protein can still bind to DNA, but is deficient in the insulator and transcription functions (59). Figures 2(A) and (B) show that the enrichment of the Myc-tagged-Tbf1Δi protein described in (49) at the interstitial Htel repeat is almost the same (8-fold) as that of the Myc-tagged Tbf1 protein.

Tbf1 forms a strong complex with two proteins containing a putative BED zinc-finger domain, Vid22 and Env11 (Ygr071c) (60, 61). Both of these proteins were shown to colocalize with Tbf1 at subtelomeric regions and promoters of some non-sno-RNA genes, where Tbf1 contributes to nucleosome displacement (62). Vid22 in a complex with Tbf1 is also involved in resection at HO-induced DSBs that are repaired by nonhomologous repair pathway (NHEJ) (63, 64). Recently, it was also demonstrated that Vid22 protein binds to and stabilizes G4-DNA forming sequences within the yeast genome (65). Note that these observations are at odds with earlier data showing that Vid22 is a plasma membrane protein required for the fructose-1,6-bisphosphatase degradation pathway (66, 67), while Env11 is involved in lysosomal vacuole functioning (68).

To confirm that Vid22 is indeed bound to the Htel repeat in our cassette, we conducted ChIP analysis for the Myc-tagged Vid22 protein. Figures 2(A) and (C) show that, similarly to the Tbf1, it is strongly (11-fold) enriched at the repeat as compared to nonrepetitive DNA. In the *tbf1Δi* genetic background, enrichment of the Vid22 protein is quite similar (10-fold). These data demonstrate that Vid22 can bind to the repeat independently of the insulator domain of the Tbf1 protein, consistent with the results in (65).

We then analyzed the role of the Tbf1 and Vid22 proteins in the instability of interstitial Htel repeats by conducting the fluctuation test experiments described above. One can see from Fig. 2(D) that the addition of the 13xMyc tag to the C-terminal end of the Tbf1 protein decreases the rate of the (CCCTAA)_38_ repeat expansion by 18-fold, suggesting that just tagging of the protein decreases its ability to promote repeat expansions. Remarkably, the strain carrying the *tbf1Δi-13xMyc* allele demonstrated a drastic 150-fold decrease in the repeat instability as compared to the *wt TBF1* strain (Fig. 2D). We, therefore, conclude that Tbf1 protein and specifically its insulator domain is responsible for the Htel repeat expansions. Deletion of *VID22* decreased the expansion rate of (CCCTAA)_38_ repeat 113-fold, *i.e*. similarly to the *tbf1Δi-13xMyc* mutation (Fig. 2D). In contrast, deletion of *ENV11* gene only mildly affects the rate of Htel repeat expansions (Figure S3, Supplementary Material). To assess genetic interactions between *TBF1* and *VID22* genes, we created a double *tbf1Δi-13xMyc vid22Δ* mutant. The rate of repeat expansions in this double mutant was decreased 224-fold. Note that the differences in rates of the repeat expansions between single and double *tbf1* and *vid22* mutants are not statistically significant. This points to the epistatic interactions of the *TBF1* and *VID22* genes when it comes to the Htel repeat instability, albeit additive interactions cannot be ruled out at present.

To estimate which class of expansions is affected most in the *tbf1* and *vid22* mutants, we analyzed the repeat size among 5-FOA^R^ clones (Figure S4, Supplementary Material). While all classes of repeat expansions had to decrease in those mutants to account for the dramatic rate decrease shown in Fig. 2(D), small-scale expansions were affected the most: down to ∼20%.

Altogether, these data suggest that it is not the binding of individual Tbf1 and Vid22 proteins to the Htel repeat, but rather their interaction, likely via the Tbf1 insulation domain, that triggers repeat expansions.

### Tbf1 and Vid22 cause a roadblock to DNA replication through the repeat

Previous research on replication fork progression through different structure-forming sequences and protein barriers has shown that Htel repeats stall replication fork in yeast (69). Here, we cloned (CCCTAA)_60_ repeats into the multicopy pRS425 plasmid with the C-rich strand as the lagging strand template for replication. This plasmid was transformed into the *wt* strain or into the mutant strains that showed decreased repeat expansions: *tbf1Δi* or *vid22Δ*. Replication fork progression through the repeat was then analyzed using 2D gel electrophoresis of the replication intermediates as described in Materials and Methods and Supplemental Material.

**Fig. 3.**
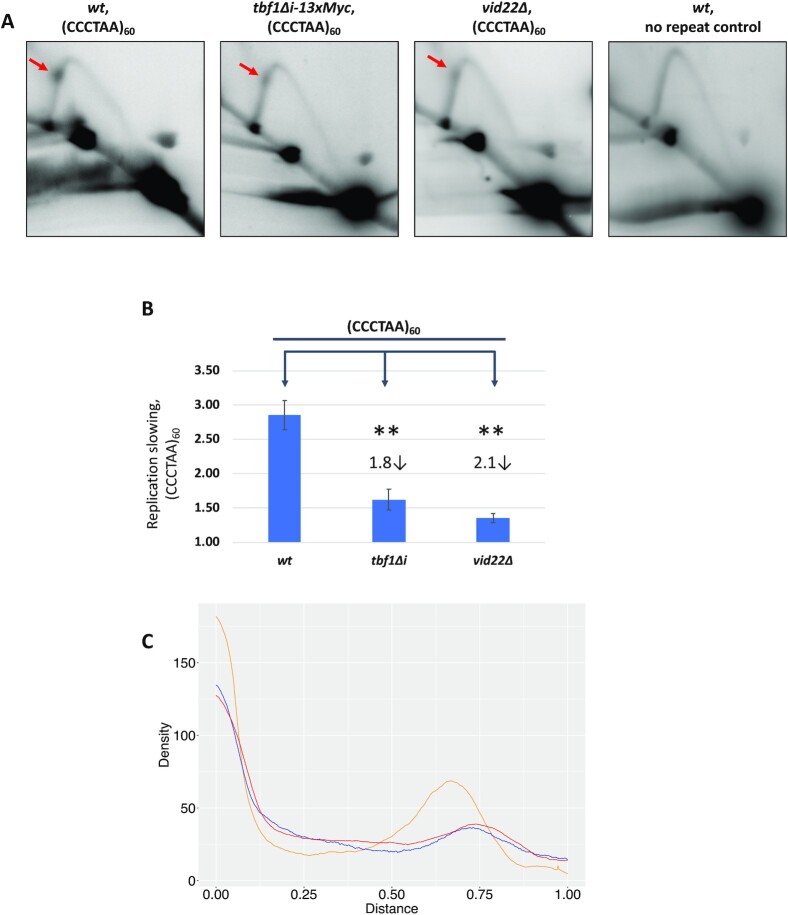
Analysis of replication intermediates with 2D gel electrophoresis. (A) Representative 2D gels of replication through (CCCTAA)_60_ repeat in *wt, tbf1Δi-13xMyc*, and *vid22Δ* strains. The strain without the repeat was used as a control. Red arrows point to the location of the (CCCTAA)_60_ repeat. (B) Quantification of replication fork slowing in *wt, tbf1Δi-13xMyc*, and *vid22Δ* strains. The ratio of radioactivity in the peak area to that corresponding area of a smooth replication arc reflects the extent of replication slowing. Numbers above the bars indicate fold of replication slowing at the (CCCTAA)_60_ repeat compared to the corresponding strain with no repeats. **—indicate significance at alpha level 0.01. SEM for three or more independent experiments are shown as error bars. (C) Densitometric profiles corresponding to the descending Y-arc region, where the (CCCTAA)_60_ repeat is located; peaks on densitograms correspond to bulges on the Y-arcs. Orange, blue, and red lines represent *wt, tbf1Δi-13xMyc*, and *vid22Δ* strains, respectively. Densitometry methods are described in Materials and Methods and Supplemental Material.

Analysis of plasmid replication in the *TBF1, tbf1Δi*, and *vid22Δ* strains is shown in Fig. 3. The presence of the long Htel repeat slows the replication fork progression down ∼3-fold, as compared to the no-repeat plasmid (Fig. 3B and C). This is, in fact, one of the strongest fork stalling signals that we have observed. In the *tbf1Δi* strain, fork stalling at the repeat is 1.8-fold weaker than in the *wt*. Since Tbf1 binding is not significantly affected by the *tbf1Δi* mutation, we conclude that it is not DNA binding per se, but the presence of its insulator domain that makes fork stalling at the repeat particularly strong.

Fork stalling at the repeat is similarly (2.1-fold) decreased in the absence of Vid22 protein (Fig. 3). Note, however, the presence of the Vid22 protein at the Htel repeat in the *tbf1Δi* mutant, is insufficient to cause strong replication stalling. These results support the idea that a complex formed by the Tbf1 and Vid22 proteins causes strong replication stalling ultimately leading to the expansions of the Htel repeat.

### Role of DNA replication in Htel repeat expansions

Previous research on the genetic control of Ytel repeat expansions implicated homologous recombination and postreplication repair (46). Thus, we looked at the effects of recombination and repair genes on expansions of the Htel (CCCTAA)_38_ repeat. Contrary to our observations in the Ytel repeat, strains carrying the *rad52Δ, sgs1Δ, rad5Δ*, and *rad6Δ* deletions showed no significant difference in the Htel repeat expansions relative to the *wt* strain (Figure S5A, Supplementary Material). A mild (4-fold) increase in the expansion rate relative to the *wt* strain was observed in *tof1Δ* and *srs2Δ* deletions (Figure S5A, Supplementary Material). Notably, in the case of Ytel repeats, both *tof1Δ* and *srs2Δ* deletions resulted in a decrease in the expansion rates (46). Mre11 is a key enzyme in DNA double-strand break repair and homologous recombination. Htel expansion rate in *mre11Δ* was not significantly different from *wt*. Thus, the genetic control of Htel repeat expansion is radically different from that of the Ytel repeat.

The Tof1 protein is a component of the fork-stabilizing complex (70). Our data that its inactivation destabilizes the Htel repeat confirms that steady replication fork progression through the repeat is needed for its stability. Mrc1 is also a part of fork-stabilizing complex, but unlike Tof1, the Mrc1 protein is not required for replication fork pausing (71). Deleting the *MRC1* gene had no effect on expansion rate of Htel repeats. We hypothesized, therefore, that replication slippage, which is known to account for the instability of numerous microsatellites (72), might destabilize Htel repeats as well.

**Fig. 4.**
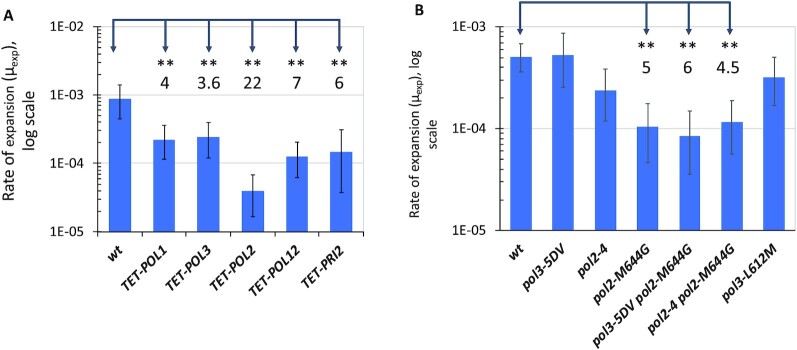
Influence of the DNA replication on the Htel repeat expansion rate. (A) Expansion rates of (CCCTAA)_38_ repeat in strains with downregulated levels of the Pol2 and Pol3 proteins (strains YL31 and YL7) as well as in strains with downregulated levels of proteins constituting the DNA polymerase alpha-primase complex Pol1, Pol12, and Pri2 (strains YL3, YL36, and YL42, respectively) when these proteins were encoded by TET-alleles of the corresponding genes (73). *URA3-Intron* cassette with (CCCTAA)_38_ repeats was inserted into these strains as well as into the isogenic YL1 *wt* strains and Htel expansion rates were evaluated. Numbers above the bars indicate fold change in expansion rate compared to the isogenic *wt* (YL1). **—nonoverlapping 95% CI compared to *wt*. (B) Influence of mutations in the exonuclease domain and nucleotide-binding pocket of the DNA polymerase epsilon and DNA polymerase delta on the expansion rate of the (CCCTAA)_38_ repeat. Strains carrying mutations *pol3-5DV* (exo- Pol δ), *pol2-4* (exo- Pol ε), *pol2-M644G* (A-motif Pol ε), *pol3-L612M* (A-motif Pol δ), double *pol3-5DV pol2-M644G*, and *pol2-4 pol2-M644G* are derivatives of ΔI(-2)I-7B-YUNI300 (51). *URA3-Intron* cassette with (CCCTAA)_38_ repeats was inserted into these strains and Htel expansion rate was evaluated. Numbers above the bars indicate fold change in expansion rate in *pol2-M644G*, double *pol3-5DV pol2-M644G*, and double *pol2-4 pol2-M644G* strains compared to the isogenic *wt* (ΔI(-2)I-7B-YUNI300 strain). **—nonoverlapping 95% CI compared to the *wt*.

To address this possibility, we first measured expansion rates of the (CCCTAA)_38_ repeat in strains in which ORFs of DNA polymerases epsilon and delta (encoded by *POL2* and *POL3* genes, respectively) and polymerase alpha-primase (encoded by *POL1, POL12*, and *PRI2* genes) were placed under the control of the TET-promoter (“TET-mutant alleles”) (73). We and others have previously found that TET-mutant alleles are recessive, and the expression of the corresponding genes is dysregulated (73, 74). Figure 4(A) shows the rate of repeat expansions was decreased in all these TET-mutants, the strongest (22-fold) inhibition was observed in TET-*POL2* mutant. This data supports the replicative model for Htel repeat expansions, specifically implicating leading strand synthesis by DNA polymerase epsilon. The profound decrease in repeat expansions in the hypomorph TET-*POL2* mutant could be caused by the exchange of Pol epsilon for Pol delta at the replication fork.

We then analyzed several missense mutations in the genes encoding for the catalytic subunits of replicative DNA polymerases epsilon and delta. Mutations *pol2-4* and *pol3-5DV* abolish the proofreading activity of DNA polymerases epsilon and delta, respectively, resulting in a mutator phenotype (75, 76). Mutations *pol2-M644G* and *pol3-L612M* are within the conservative A-motif of the corresponding DNA polymerases, which is essential for dNTP selection in the polymerases’ active sites (77–80). DNA polymerases carrying these mutations retain robust polymerization activity, but their DNA synthesis fidelity is reduced (77–81). Importantly, the signature of Pol2-M644G leading strand polymerase is misincorporation of dTTP opposite to T in the DNA template. Since in our case, the leading strand template contains the TTAGGG repeat, misincorporation of T opposite to T within the repetitive run is expected to decrease its ability for slippage. The effects of different DNA polymerase mutations on expansions of the (CCCTAA)_38_ repeat within the *URA3-Intron* reporter are shown in Fig. 4(B). Clearly, defects in the proofreading activity of DNA polymerases epsilon and delta do not affect Htel repeat expansions (Fig. 4B). At the same time, repeat expansion rates are significantly decreased by the *pol2-M644G* mutation, but not by the *pol3-L612M* mutation (Fig. 4B).

DNA polymerase epsilon holoenzyme has four subunits, two of which—the small accessory subunits Dpb3 and Dpb4–contribute its high processivity (82, 83). Dpb3 and Dpb4 subunits also have histone-fold motifs, which contribute to DNA polymerase epsilon's ability to bind double-stranded and single-stranded DNA and interact with nucleosomes (84). We evaluated the expansion rate of (CCCTAA)_38_ tract in strains, which carry single and double deletions of the *DPB3* and *DPB4* genes and found that double deletion of the *DPB3* and the *DPB4* genes elevated expansion rate of the (CCCTAA)_38_ repeat about 4-fold (Figure S5B, Supplementary Material), further emphasizing the role of DNA Pol epsilon processivity in the expansion of the Htel repeats.

Altogether, these results suggest that leading strand DNA polymerase epsilon might contribute to Htel repeat expansions to a larger extent than DNA polymerase delta. Further studies are needed to understand differential contribution of replicative DNA polymerases to Htel repeat expansion.

## Discussion

We have demonstrated that Htel (TTAGGG)_n_•(CCCTAA)_n_ placed inside a yeast chromosome III demonstrate a strong propensity to expand. An Htel tract, consisting of (CCCTAA)_38_ repeats, expands at a rate of ∼4 × 10^–4^ per cell per generation. We observed small-, mid-, and large-scale expansions, and in some cases a repetitive tract can double in one step.

Previously, we have found that Ytel repeats placed inside the same chromosome III were also prone for expansions. Expansions of these (TGTGTGGG)_n_ tracts were driven by homologous recombination and/or postreplication DNA repair. Expansions of Htel repeats at the same location, in contrast, did not depend on either of these processes. Thus, while phenomenologically similar, expansions of Ytel and Htel repeats rely on different mechanisms.

In *Saccharomyces cerevisiae*, the Tbf1 protein is known to bind to subtelomeric TTAGGG-like STAR-repeats preventing heterochromatin spreading from telomeres inward (42), i.e. working as an insulator. The Tbf1 insulator domain is important for its transactivation activity and prevention of silencing (59). The *tbf1Δi* mutation leads to a defect in length regulation of artificial telomeres built of TTAGGG-repeat, indicating that the insulator domain of Tbf1 is required for regulation of telomere length (49). Using ChIP, we found that Tbf1 is strongly enriched at interstitial Htel repeats. Tbf1Δi protein is also clearly enriched at the repeat, owing to the fact that its DNA-binding domain lies outside of the insulator domain. Tbf1 has a physical and functional partner, Vid22, which was implicated in transcription of certain genes, binding to G4-motifs, and R-loop destabilization (62, 64, 65). Our ChIP analysis showed a strong presence of the Vid22 protein at the interstitial Htel repeat both in the *TBF1* and the *tbf1Δi* genetic background (Fig. 2A and C).

We then looked at how mutations in *TBF1* and *VID22* genes affect Htel repeat expansions. Figure 2(D) shows that deletion of the Tbf1 insulator domain results in a dramatic stabilization of interstitial Htel repeats. Adding a Myc-tag to Tbf1 also leads to a decreased expansion rate. The Myc-tag is located adjacent to the C-terminal DNA binding domain of Tbf1. It does not preclude specific protein–DNA binding, as we see enrichment of the Tbf1-13xMyc at the repeat (Fig. 2A and B). It is possible, however, that the tag might decrease Tbf1-DNA binding somewhat, which results in a lower expansion rate. *VID22* deletion leads to a similarly dramatic repeat stabilization. The repeat stabilization in a double *tbf1Δi vid22Δ* mutant is slightly stronger, but this difference is not statistically significant from the single mutant, i.e. the two mutations are likely epistatic to each other. Note, however, that the *tbf1Δi* mutation does not affect the Vid22 binding to the repeat and it prevents repeat expansions regardless of the presence of the Vid22 protein. We believe that *VID22* deletion does not prevent Tbf1 binding to its target sequence, since this strain is perfectly viable, as opposed to the *TBF1* deletion. Altogether the ChIP and instability data suggest that a complex of the Tbf1 and Vid22 proteins, likely stabilized by the Tbf1 insulator domain, is at heart of the Htel repeat expansions.

Addition of extra repeats to the repetitive tract must require DNA synthesis. For previously studied Ytel repeats, we have found that DNA synthesis that occurs during DNA recombination or postreplication repair is responsible for their expansions (46). In case of the Htel repeat, however, proteins involved in homologous recombination or postreplication repair have no bearing on its expansion (Figure S5A, Supplementary Material).

An alternative mechanism for repeat expansions, which is widely discussed in the literature (72, 85), is the formation of slip-outs on nascent DNA strands during replication. In this case, a bumpy progression of the replication fork through the interstitial Htel repeat accompanied by nascent strand slippages would cause repeat expansions. Three groups of our data support this hypothesis. First, replication fork progression is indeed stalled at the Htel repeat (Fig. 3), while *tbf1Δi* and *vid22Δ* mutations, which preclude repeat expansions, weaken fork stalling. Second, various mutations in the replicative DNA polymerases stabilize Htel repeats. Notably, mutations in the leading strand DNA polymerase epsilon had the strongest effects. Third, deletion of the *TOF1* gene, encoding a key component of the fork stabilizing complex, increases repeat expansions. The fork stabilizing complex links the replicative CMG helicase with DNA polymerase epsilon and is vital for the smooth replication fork progression (86, 87). Our data on the scale of repeat expansions for (CCCTAA)_38_ repeat in the *wt* strain and *tbf1Δi* and *vid22Δ* mutants is in-line with our slippage hypothesis: the majority of repeat expansion in the *wt* strain are small-scale (Fig. 1E), and they are the most affected by the *tbf1Δi* and *vid22Δ* mutations (Figure S4, Supplementary Material).

Altogether, we hypothesize that replication fork progression through the Htel repeat is impeded by the Tbf1/Vid22 protein complexes, which leads to strand slippage during DNA synthesis and, ultimately, repeat expansions. The repetitive slip-outs can either be formed on the leading strand synthesized by DNA polymerase epsilon. Alternatively, impeded progression of DNA polymerase epsilon could provoke the formation of slip-outs during the lagging strand synthesis.

Very recently, Galati et al. (65) showed that the Vid22 protein binds to G4-containing regions in yeast genome and prevents chromosomal fragility and GCRs at those regions. In our case, however, we see that Vid22 promotes expansions of Htel that are known to form G4-DNA (9). What could be the reasons for these differences? The first possibility is that the G4-forming repeats studied in Galati et al. (65) are short, as compared to our fairly long Htel. It is foreseeable, therefore, that strong protein barriers formed by multiple Tbf1/Vid22 pairs at long Htel repeats are far more detrimental for their replication and stability than their ability to form G4-DNA. Alternatively, one can imagine that stabilization of G4 slip-out on nascent DNA strand by the Tbf1/Vid22 complex could result in repeat expansions.

The latter scenario looks somewhat similar to the stabilization of triplet repeat slip-outs by the MutSβ mismatch repair complex in mammalian cells, which ultimately promotes repeat expansions (88). Note that the role of MutSβ in repeat expansions in yeast is far less obvious: it promotes mid-range expansions of CAG repeats in some studies (89, 90), but has no effect or even decreases them in other studies (91–93). Further, it has no effect on (GAA)_n_ repeat expansions (57) and only a minor effect on (GAA)_n_ contractions (94). Aside from expandable trinucleotide repeats, MMR stabilizes mono- and dinucleotide repeats in yeast and humans, by preventing their slippage (95, 96). Future studies are warranted to analyze the role of the mismatch repair system in the Tbf1/Vid22-dependent instability of the Htel repeats.

Could our yeast data help the understanding of instability of interstitial telomeric repeats in human cells? Human orthologs of the Tbf1, TRF1, and TRF2, are known to bind ITSs and recruit additional proteins (11, 29, 35, 38). Polyclonal antibodies raised against Tbf1 C-terminal binding domain were able to recognize human TRF1 and TRF2 proteins confirming that Tbf1 and TRF1/TRF2 are immunologically related (44). Human orthologs of the Vid22 protein are not known. There is, however, a human protein, that contains the BED-finger domain as Vid22, called ZBED1/hDREF (61, 97). ZBED1 is a transcription factor, and potential ZBED1 binding sites are found in the promoter regions of many genes involved in DNA replication and repair, cell cycle regulation, chromatin remodeling, and transcription (98, 99). Transcription of ZBED1 is elevated is G1-S Phase of the cell cycle and instead, reduction of ZBED1 protein resulted in inhibition of G1-S progression (99). Increased expression of ZBED1 in gastric cancer tissues leads to proliferation and apoptosis of tumor cells (100). This is particularly interesting with regard to our results, since length polymorphism of interstitial telomeric repeats was seen in gastric cancers (30, 101). Therefore, ZBED1 may be a novel therapeutic target and it would be of great interest to study the role of the ZBED1 protein in the integrity of interstitial telomeric repeats in human genome.

## Supplementary Material

pgac080_Supplemental_File

## Data Availability

All data are presented within the manuscript or are available in the Supplementary Material.
