# Supplementary material for: Partners in crime: Tbf1 and Vid22 promote expansions of long human telomeric repeats at an interstitial chromosome position in yeast
Source: PNAS Nexus. 2022 Jun 8;1(3):pgac080. doi: 10.1093/pnasnexus/pgac080 (PMC9272169; doi:10.1093/pnasnexus/pgac080)
Supplement: pgac080_Supplemental_File [file pgac080_supplemental_file.pdf]

Supplementary material

Construction of HTel plasmids in the *URA3* cassette

To clone Htel repeats we used the derivative of pRS315-ADE2-IntGAA120 plasmid (1), which contained *ACT1* intron with $(GAA)_{120}$ repeats inserted into the StuI site of the pRS315-ADE2 plasmid. The insert carrying the intron and GAA repeats was obtained by PCR with primers IntrF and IntrR (anneal at the 5' and 3' borders of the *ACT1* intron) from the SMY221 strain (2). This plasmid we used to generate the pRS315-ADE2-Lint2 plasmid which omitted the GAA repeats but contained the Lint adapter within the *ACT1* intron for easy cloning of various sequences into it. This plasmid was obtained by digestion of the pRS315-ADE2-IntrGAA120 plasmid with BsaBI and SphI and inserting the Lint adapter (one side - blunt and another - SphI site) obtained by annealing two oligos Lint1 and Lint2. The resultant polylinker comprised restriction sites for BssHI, AvrII, StuI, NruI, FbaI, BalI, NcoI, SphI.

Human telomeric tracts of varying length were generated by annealing the oligonucleotides HTU and HTD, and then ligating the resultant duplexes with T4 ligase (Thermo). The ligation products with recessed 3'-termini were filled in with either Klenow fragment (Thermo) or T4 DNA polymerase (Thermo) to generate blunt ends. The resultant products were ligated into the NruI site of the pRS315-ADE2-Lint2 plasmid and the ligation mix was transformed into XL10-Gold competent cells. Colonies with Htel inserts were selected and sequenced to determine the length of the repetitive tract, its orientation, and its integrity. We generated plasmid pRS315-ADE2-Lint2-BHT1 with $(TTAGGG)_{25}$ repeats, plasmid pRS315-ADE2-Lint2-NBHT44 with $(CCCTAA)_{32}$ repeats, plasmid pRS315-ADE2-Lint2-BHT25 with $(TTAGGG)_{39}$ repeats and plasmid pRS315-ADE2-Lint2-BHT7 with $(CCCTAA)_{60}$ repeats. The inserts containing tracts of Htel repeats were cut from these plasmids with StuI and MscI (both generate blunt ends) and were inserted into the blunted XhoI site of the pISL-UR-Intron-A3-TRP1-ISR plasmid (aka UIRL) (3). Since blunt end ligation allows the sequence to be inserted in both orientations, we produced UIRL plasmid derivatives with 25, 32, 39 and 60 Htel repeats in G-orientation (UIRL-BHT1-9, UIRL-NBHT44-12, UIRL-BHT25-11, and UIRL-BHT7-19, respectively) and 25, 32, 38 and 60 repeats in C-orientation (UIRL-BHT1-11, UIRL-NBHT44-18, UIRL-BHT25-12, and UIRL-BHT7-18, respectively). The integrity of the repeat as well as the *URA3-Intron* gene was evaluated by sequencing.

Plasmids used for two-dimensional electrophoresis were constructed using the pRS425 plasmid. The $(CCCTAA)_{60}$ repeat and the control no-repeat sequence were amplified from the UIRL- BHT7-18 or UIRL plasmids using primers UIRL1-BsrGI and UIRL2-BsrGI, which were then inserted into the BsrGI restriction site via ligation. The resultant pRS425-7-18B and pRS425-UIRLB plasmids were sequenced and used for yeast transformation.

2D-gel quantification

Quantification of 2D gels was performed with NIH ImageJ program and a custom R-script (https://github.com/senselle/2D-gel-quantification). First, the figures obtained on the Amersham Typhoon scanner were quantified in ImageJ as follows: a wide segmented line was drawn from the 2x spot to the 1.5x spot to include the whole descending arc. Then the data from the plot of this line were saved as an csv file. Also, a parallel line was drawn above and below the arc to estimate the background noise. The data from these two plots were added to the file with arc. The resultant csv file contained columns A-B with the data belonging to the arc, columns C-D with the data belonging to the background noise above and then E-F columns with

the data of the background noise below the arc. This file was then opened with the RStudio and a plot was made using ggplot. The plot was then opened with the ImageJ software. The lines were drawn as shown in (4) and the peak area and the corresponding area of a smooth replication arc were measured to calculate the replication fork slowing.

References

1. R. J. McGinty *et al.*, A Defective mRNA Cleavage and Polyadenylation Complex Facilitates Expansions of Transcribed (GAA)n Repeats Associated with Friedreich's Ataxia. *Cell Rep* **20**, 2490-2500 (2017).
2. A. A. Shishkin *et al.*, Large-scale expansions of Friedreich's ataxia GAA repeats in yeast. *Mol. Cell* **35**, 82-92 (2009).
3. A. Y. Aksenova *et al.*, Genome rearrangements caused by interstitial telomeric sequences in yeast. *Proc. Natl Acad. Sci. USA* **110**, 19866-19871 (2013).
4. M. M. Krasilnikova, S. M. Mirkin, Analysis of triplet repeat replication by two-dimensional gel electrophoresis. *Methods Mol Biol* **277**, 19-28 (2004).
5. Y. I. Pavlov, C. S. Newlon, T. A. Kunkel, Yeast origins establish a strand bias for replicational mutagenesis. *Mol. Cell* **10**, 207-213 (2002).
6. V. Ribaud, C. Ribeyre, P. Damay, D. Shore, DNA-end capping by the budding yeast transcription factor and subtelomeric binding protein Tbf1. *EMBO J.* **31**, 138-149 (2012).
7. Y. Zhang *et al.*, Genome-wide screen identifies pathways that govern GAA/TTC repeat fragility and expansions in dividing and nondividing yeast cells. *Mol. Cell* **48**, 254-265 (2012).
8. Anna Y. Aksenova, G. Han, Alexander A. Shishkin, Kirill V. Volkov, Sergei M. Mirkin, Expansion of Interstitial Telomeric Sequences in Yeast. *Cell Reports* **13**, 1545-1551 (2015).

Table S1. Strains constructed in this study.

Name	Description	Comments
SMY706	*MATa leu2-1 trp1-63 ura3-52 his3-200*	(3)
SMY710	*MATa leu2-1 trp1-63 ura3-52 his3-200 ade2Δ::KanMX4*	(3)
SMY803	*MATa leu2-1 trp1-63 ura3-52 his3-200 ade2Δ::KanMX4 III (75423-75715)::URA3-Intron*	(3)
SMY716	Same as SMY710 *III (75423-75715)::URA3-Intron*-$(TTAGGG)_{25}$	Insertion of $(TTAGGG)_{25}$ into the *URA3-Intron*
SMY717	Same as SMY710 *III (75423-75715)::URA3-Intron*-$(CCCTAA)_{25}$	Insertion of $(CCCTAA)_{25}$ into the *URA3-Intron*
SMY718	Same as SMY710 *III (75423-75715)::URA3-Intron*-$(CCCTAA)_{60}$	Insertion of $(CCCTAA)_{60}$ into the *URA3-Intron*
SMY719	Same as SMY710 *III (75423-75715)::URA3-Intron*-$(TTAGGG)_{60}$	Insertion of $(TTAGGG)_{60}$ into the *URA3-Intron*
SMY757	Same as SMY710 *III (75423-75715)::URA3-Intron*-$(TTAGGG)_{39}$	Insertion of $(TTAGGG)_{39}$ into the *URA3-Intron*
SMY758	Same as SMY710 *III (75423-75715)::URA3-Intron*-$(CCCTAA)_{38}$	Insertion of $(CCCTAA)_{38}$ into the *URA3-Intron*
SMY759	Same as SMY710 *III (75423-75715)::URA3-Intron*-$(TTAGGG)_{32}$	Insertion of $(TTAGGG)_{32}$ into the *URA3-Intron*
SMY760	Same as SMY710 *III (75423-75715)::URA3-Intron*-$(CCCTAA)_{32}$	Insertion of $(CCCTAA)_{32}$ into the *URA3-Intron*
YIP1	*MAT***a** *CAN1 his7-2 leu2Δ::kanMX ura3Δ trp1-289 ade2-1 lys2-ΔGG2899-2900*	ΔI(-2)I-7B-YUNI300 (5)
YIP2	Same as YIP1 *pol3-5DV*	Exonuclease-deficient Pol δ
YIP3	Same as YIP1 *pol3-5DV pol2-M644G*	Exonuclease-deficient Pol δ plus mutation in the A-motif of Pol2
YIP5	Same as YIP1 *pol2-4*	Exonuclease-deficient Pol ε
YIP6	Same as YIP1 *pol2-4 pol2-M644G*	Exonuclease-deficient Pol ε plus mutation in the A-motif of Pol2
YIP7	Same as YIP1 *pol2-M644G*	Mutation in the A-motif of Pol2
AAY18	Same as YIP1 *III (75423-75715)::URA3-Intron*-$(CCCTAA)_{38}$	Insertion of the *URA3-Intron*-$(CCCTAA)_{38}$ cassette
AAY19	Same as YIP2 *III (75423-75715)::URA3-Intron*-$(CCCTAA)_{38}$	Insertion of the *URA3-Intron*-$(CCCTAA)_{38}$ cassette
AAY20	Same as YIP5 *III (75423-75715)::URA3-Intron*-$(CCCTAA)_{38}$	Insertion of the *URA3-Intron*-$(CCCTAA)_{38}$ cassette
AAY21	Same as YIP6 *III (75423-75715)::URA3-Intron*-$(CCCTAA)_{38}$	Insertion of the *URA3-Intron*-$(CCCTAA)_{38}$ cassette
AAY25	Same as YIP3 *III (75423-75715)::URA3-Intron*-$(CCCTAA)_{38}$	Insertion of the *URA3-Intron*-$(CCCTAA)_{38}$ cassette

AAY26	Same as YIP7 *III (75423-75715)::URA3-Intron*-$(CCCTAA)_{38}$	Insertion of the *URA3-Intron*-$(CCCTAA)_{38}$ cassette
AAY39	Same as YIP1 *pol3-L612M*	Mutation in the A-motif of Pol3
AAY40	Same as AAY39 *III (75423-75715)::URA3-Intron*-$(CCCTAA)_{38}$	Insertion of the *URA3-Intron*-$(CCCTAA)_{38}$ cassette
AAY30	Same as SMY758 *dpb3Δ*	*DPB3* is deleted using PCR product obtained from pRS303 plasmid using oligos DPB3FHIS and DPB3RHIS
AAY31	Same as SMY758 *dpb4Δ*	*DPB4* is deleted using PCR product obtained from pRS303 plasmid using oligos DPB4FHIS and DPB4RHIS
AAY42	Same as AAY31 *dpb3Δ*	*DPB3* is deleted using PCR product obtained from pUC19-HphMX4 plasmid using oligos DPB3FHIS and DPB3RHIS
AAY53	Same as SMY758 *rad52Δ*	*RAD52* is deleted using PCR product obtained from pAG32 plasmid using oligos KR52t-F and KR52h-R
AAY54	Same as SMY758 *rad5Δ*	*RAD5* is deleted using PCR product obtained from pAG32 plasmid using oligos RAD5/SP and RAD5/ASP
AAY55	Same as SMY758 *tof1Δ*	*TOF1* is deleted using PCR product obtained from pAG32 plasmid using oligos TOF1/SP and TOF1/ASP
AAY58	Same as SMY758 *mre11Δ*	*MRE11* is deleted using PCR product obtained from pRS303 plasmid using oligos MRE11FCHIS and MRE11RCHIS
AAY60	Same as SMY758 *rad6Δ*	*RAD6* is deleted using PCR product obtained from pRS303 plasmid using oligos RAD6S-His and RAD6R-His
AAY61	Same as SMY758 *srs2Δ*	*SRS2* is deleted using PCR product obtained from pRS303 plasmid using oligos SRS2FHIS and SRS2RHIS
AAY62	Same as SMY758 *sgs1Δ*	*SGS1* is deleted using PCR product obtained from pRS303 plasmid using oligos SGS1FCHIS and SGS1RCHIS
AAY65	Same as SMY758 *mrc1Δ*	*MRC1* is deleted using PCR product obtained from pRS303 plasmid using oligos MRC1DFHIS3 and MRC1DRHIS3
YVR032	W303 *(RAD5, lys2), mata::loxP, leu2::pGALHO, mnt2::LYS2 TBF1*-13Myc-His3MX6 Chr. VII-L::*ADE2*-Dbp(amp7)-200 bp phage lambda DNA-T2AG3-60-HO site, Chr. V-	(6)

	R::*TRP1*-Dbp(amp9)-200 bp phage lambda DNA-TG-80-HO site	
YVR118	W303 *(RAD5, lys2), mata::loxP, leu2::pGALHO, mnt2::LYS2 tbf1-Δi*-13Myc-His3MX6 Chr. VII-L::*ADE2*-Dbp(amp7)-200 bp phage lambda DNA-T2AG3-60-HO site, Chr. V-R::*TRP1*-Dbp(amp9)-T2AG3-230-HO site	(6)
SMY906	Same as SMY803 *TBF1-13xMyc-HIS3MX6*	*TBF1-13xMyc-HIS3MX6* is integrated using PCR product obtained from YVR032 strain using oligos TBF1-wt-CMyc-F and TBF1-CMyc-R
SMY904	Same as SMY758 *TBF1-13xMyc-HIS3MX6*	*TBF1-13xMyc-HIS3MX6* is integrated using PCR product obtained from YVR032 strain using oligos TBF1-wt-CMyc-F and TBF1-CMyc-R
SMY903	Same as SMY803 *tbf1Δi-13xMyc-HIS3MX6*	*tbf1Δi-13xMyc-HIS3MX6* is integrated using PCR product obtained from YVR118 strain using oligos TBF1-di-CMyc-F and TBF1-CMyc-R
SMY901	Same as SMY758 *tbf1Δi-13xMyc-HIS3MX6*	*tbf1Δi-13xMyc-HIS3MX6* is integrated using PCR product obtained from YVR118 strain using oligos TBF1-di-CMyc-F and TBF1-CMyc-R
YL1	*MATa, bar1-Δ, trp1-Δ, his3-Δ, ura3-Δ, leu2-Δ, ade2-Δ, lys2-Δ, met15-Δ, V34205::ADE2*	(7)
YL3	Same as YL1 *TET-POL1*	(7)
YL7	Same as YL1 *TET-POL3*	(7)
YL31	Same as YL1 *TET-POL2*	(7)
YL36	Same as YL1 *TET-POL12*	(7)
YL42	Same as YL1 *TET-PRI2*	(7)
SMY845	Same as YL1 *III (75423-75715)::URA3-Intron*-$(CCCTAA)_{38}$	Insertion of the *URA3-Intron*-$(CCCTAA)_{38}$ cassette
SMY846	Same as YL3 *III (75423-75715)::URA3-Intron*-$(CCCTAA)_{38}$	Insertion of the *URA3-Intron*-$(CCCTAA)_{38}$ cassette
SMY847	Same as YL7 *III (75423-75715)::URA3-Intron*-$(CCCTAA)_{38}$	Insertion of the *URA3-Intron*-$(CCCTAA)_{38}$ cassette
SMY848	Same as YL31 *III (75423-75715)::URA3-Intron*-$(CCCTAA)_{38}$	Insertion of the *URA3-Intron*-$(CCCTAA)_{38}$ cassette
SMY849	Same as YL36 *III (75423-75715)::URA3-Intron*-$(CCCTAA)_{38}$	Insertion of the *URA3-Intron*-$(CCCTAA)_{38}$ cassette
SMY850	Same as YL42 *III (75423-75715)::URA3-Intron*-$(CCCTAA)_{38}$	Insertion of the *URA3-Intron*-$(CCCTAA)_{38}$ cassette

SMY1071	Same as SMY758 *vid22Δ*	*VID22* is deleted using PCR product obtained from pAG25 plasmid using oligos VID22DTF and VID22DRC
SMY1072	Same as SMY758 *env11Δ*	*ENV11* is deleted using PCR product obtained from pAG32 plasmid using oligos ENV11DTF and ENV11DRC
SMY1073	Same as SMY901 *vid22Δ*	*VID22* is deleted using PCR product obtained from pAG25 plasmid using oligos VID22DTF and VID22DRC
SMY1074	Same as SMY901 *env11Δ*	*ENV11* is deleted using PCR product obtained from pAG32 plasmid using oligos ENV11DTF and ENV11DRC
SMY1075	Same as SMY758 *VID22-13xMyc-HIS3MX6*	*VID22-13xMyc-HIS3MX6* is integrated using PCR product obtained from YVR032 strain using oligos VID22_Myc_HIS3MX6_F and VID22_Myc_HIS3MX6_R
SMY1076	Same as SMY758 *tbf1Δi*	Strain SMY901 was used to delete 13xMyc tag from *tbf1Δi-13xMyc-HIS3MX* using CRISPR-Cas9 method. Oligos TBF1_MYCdel_PRCC_F and TBF1_MYCdel_PRCC_R were used to amplify the pRCC-N plasmid. Oligo TBF1_MYCdel_template was used as a repair template.
SMY1083	Same as SMY1076 *VID22-13xMyc-HIS3MX6*	*VID22-13xMyc-HIS3MX6* is integrated using PCR product obtained from YVR032 strain using oligos VID22_Myc_HIS3MX6_F and VID22_Myc_HIS3MX6_R
SMY1081	Same as SMY803 *VID22-13xMyc-HIS3MX6*	*VID22-13xMyc-HIS3MX6* is integrated using PCR product obtained from YVR032 strain using oligos VID22_Myc_HIS3MX6_F and VID22_Myc_HIS3MX6_R
SMY710 pRS425-7-18B	SMY710 with pRS425-7-18B plasmid	Used for 2D gel electrophoresis
SMY710 pRS425-UIRLB	SMY710 with pRS425-UIRLB plasmid	Used for 2D gel electrophoresis
SMY1070	Same as SMY710 *tbf1Δi-13xMyc-HIS3MX6*	*tbf1Δi-13xMyc-HIS3MX6* is integrated using PCR product obtained from YVR118 strain using oligos TBF1-di-CMyc-F and TBF1-CMyc-R

SMY1070 pRS425-7-18B	SMY1070 with pRS425-7-18B plasmid	Used for 2D gel electrophoresis
SMY1106	Same as SMY710 *vid22Δ*	*VID22* is deleted using PCR product obtained from pAG32 plasmid using oligos VID22DTF and VID22DRC
SMY1106 pRS425-7-18B	SMY1106 with pRS425-7-18B plasmid	Used for 2D gel electrophoresis

Table S2. Primers

Primer	Sequence	Comments
Intr-F	GTATGTTCTAGCGCTTGCACC	Intron cloning with repeat
Intr-R	CTAAACATATAATATAGCAACAAAAGAATGAAGCA	Intron cloning with repeat
Lint1	Phos-GCGCGCCTAGGCCTCGCGATGATCATGGCCATGGCATG	Inserting the Lint adapter
Lint2	Phos-CCATGGCCATGATCATCGCGAGGCCTAGGCGCGC	Inserting the Lint adapter
AdStu-F	CCCGGTTGTGGTATATTTGGTG	Repeat region amplification in the *ADE2-Intron* cassette
AdSu-R	GCATAATGGCGTTCGTTGTAATGG	Repeat region amplification in the *ADE2-Intron* cassette
HTU	AGGGTTAGGGTTAGGGTTAGGGTT	Htel repeat generation
HTD	CCTAACCCTAACCCTAACCCTAAC	Htel repeat generation
UIRL1	GAAGTAACAAAGGAACCTAGAGGGTA	Htel repeat amplification with flanking regions
UIRL2	TACAGATCAGTCAATATAGGAGGTT	Htel repeat amplification with flanking regions
BHT1OR1	CCCAATTGCTCGCCTCG	Htel repeat amplification in SMY716, SMY757, forward primer
BHTUNI	CTCGACCATGATCATCG	Htel repeat amplification in SMY716, SMY717, SMY757, SMY759, SMY760; reverse primer
BHT1OR2	GAGAAATCTCTCGCCTCG	Htel repeat amplification in SMY717, forward primer
BHT7OR1	CCCAATTGCTCGACCTCG	Htel repeat amplification in SMY760, forward primer
BHTUNI2	GCTCGCCATGATCATCG	Htel repeat amplification in SMY719, reverse primer
BHT7191	GAGAAATCTCTCGACCT	Htel repeat amplification in SMY719, SMY759, SMY758, forward primer
108-F	CCCAATTGCTCGACCATGATCATCGACC	Alternative primers for Htel repeat amplification in SMY717, SMY782 and its derivatives; forward primer
108-R	GGTAAAGAGAAATCTCTCGCCTCGGGTTAG	Alternative primers for Htel repeat amplification in SMY717, SMY782 and its derivatives; reverse primer

102-F	GCCATTTATTGTCGCAGTAAGGA	Forward primer used to amplify the *URA3-Intron* gene
TrpM-R	GCAAGTCAGCATCGGAATCTAGAG	Reverse primer used to amplify the *URA3-Intron* gene
UseqR	TGGTTTCTTAGACGTCGACG	*URA3* sequencing
UseqF	GATTCGGTAATCTCCGAACAG	*URA3* sequencing
UseqMF	GTATACAGAATAGCAGAATGGGCA	*URA3* sequencing
UseqMR	CATGAGACTTAGTAACAGTAGC	*URA3* sequencing
A36b-F	ACGTGTACAGTTCTCTTTACATCATC	External primer used to amplify *URA3-Intron-TRP1* cassette. Verification of the *URA3-Intron-TRP1* cassette integration; forward primer
A36a-R	AGGGTCGTTGCCTTCTGGTGTT	External primer used to amplify *URA3-Intron-TRP1* cassette. Verification of the *URA3-Intron-TRP1* cassette integration; reverse primer
2511D	GAGAAATCTCTCGACCATGATCATCGTA A	Alternative primers for Htel repeat amplification in SMY757; forward primer
2511R	GCACGGTCCCAATTGCTCGCCTCGGGGT T	Alternative primers for Htel repeat amplification in SMY757; reverse primer
MRE11FC HIS	AGCCCTTGGTTATAAATAGGATATAATA TAATATAGGGATCAAGTACAACGGTGT CGGGGCTGGCTTAA	Deleting the *MRE11* gene with the *HIS3* cassette
MRE11R CHIS	ATGCAGACAATTGACGCAAGTTGTACCT GCTCAGATCCGATAAAACTCGACTCCTT ACGCATCTGTGCGGTA	Deleting the *MRE11* gene with the *HIS3* cassette
MRE11TF C	GAGCAAAGGCTGGAAAACTAAGT	Verification of the *MRE11* deletion, used in pair with HIS3H-R
MRE11T RC	TGGCCAATCGAATAGAACCCA	Verification of the *MRE11* deletion, used in pair with HIS3T-F
RAD6S-His	CATGTCCACACCAGCTAGAAGAAGGTT GATGAGAGATTTTAAACGTATGAAGGA AGGTGTCGGGGCTGGCTTAA	Deleting the *RAD6* gene with the *HIS3* cassette
RAD6A-His	TCAGTCTGCTTCGTCGTCGTCGTCGTCG TCATCATCATCATCATCATCGTCCATCT CCTTACGCATCTGTGCGGTA	Deleting the *RAD6* gene with the *HIS3* cassette
RAD6TS	GCCGGAGTAGAAAGCTGGAA	Verification of the *RAD6* deletion, used in pair with HIS3H-R

RAD6TA	AAAGATACGGGTATCGGCAGTT	Verification of the *RAD6* deletion, used in pair with HIS3T-F
SRS2FHIS	GCACTTTGAGTATCATTCCAATTTGATCTTTCTTCTACCGGTACTTAGGGGGTGTCGGGGCTGGCTTAA	Deleting the *SRS2* gene with the *HIS3* cassette
SRS2RHIS	AAGTGCTACTAATCGATGACTATGATTTCACCGTTGTTTAATTTTGACTTCTCCTTACGCATCTGTGCGGTA	Deleting the *SRS2* gene with the *HIS3* cassette
SRS2TF	GAGTTACCTCCAGCTATCCTGA	Verification of the *SRS2* deletion, used in pair with HIS3H-R
SRS2TR	GGCATACTGCTCATTCATAGCTGT	Verification of the *SRS2* deletion, used in pair with HIS3T-F
SGS1FCHIS	CACTTTCTTCCTCTGTAGTGACCTCGGTAATTTCTAAAACCTCGTCTCCCGGTGTCGGGGCTGGCTTAA	Deleting the *SGS1* gene with the *HIS3* cassette
SGS1RCHIS	GGCGGTAATGGTGACGAAGCCGTCACATAACTTAAGAAGGGAGCACAAATCTCCTTACGCATCTGTGCGGTA	Deleting the *SGS1* gene with the *HIS3* cassette
SGS1TFC	CTGTCGCTAGACTGGATGACA	Verification of the *SGS1* deletion, used in pair with HIS3H-R
SGS1TRC	GGCACTTAGCAGGCTGGGTGA	Verification of the *SGS1* deletion, used in pair with HIS3T-F
DPB4FHIS	AAGCCTTTCATTGCTTATTTATATCAGACCATATATTTTTACACACGATGGGTGTCGGGGCTGGCTTAA	Deleting the *DPB4* gene with the *HIS3* cassette
DPB4RHIS	GGTTACGTTTGCTCAAGGTTTTGAACTCTAGTTTCTACATCTTGGCTCTCCTCCTTACGCATCTGTGCGGTA	Deleting the *DPB4* gene with the *HIS3* cassette
DPB4TF	CGGCACAATAGCAGAAGCCACA	Verification of the *DPB4* deletion, used in pair with HIS3H-R
DPB4TR	CGTTGGGTTCCCCGGCTTG	Verification of the *DPB4* deletion, used in pair with HIS3T-F
DPB3FHIS	CAACTGCCTGCCCCAACAGATAAAAACAAGCAAGGGTCAACCGTGTTGCAGGTGTCGGGGCTGGCTTAA	Deleting the *DPB3* gene with the *HIS3* cassette or hphMX4 cassette
DPB3RHIS	CAACAACACTAAGGATCGGTGCTTTTCGTATGGTCAACTTCGATATCAGACTCCTTACGCATCTGTGCGGTA	Deleting the *DPB3* gene with the *HIS3* cassette or hphMX4 cassette

DPB3TF	GCCCCTTCTACCTCAATGACA	Verification of the *DPB3* deletion, used in pair with HIS3H-R or KanH-R
DPB3TR	CTACATAACAGCTCTAACCTACA	Verification of the *DPB3* deletion, used in pair with HIS3T-F or KanT-F
RAD5/SP	CACAATATGAGTCATATTGAACAGGAAGAAAGGAAGAGGTTTTTAACGATGACCCAGCTGAAGCTTCGTACGC	Deleting the *RAD5* gene with the hphMX4 cassette
RAD5/ASP	CATCTTTTGAGAAAGTATGAGTTAGCTCTTTCTCCAGGATATCCAAGTATGTAGACATAGGCCACTAGTGGATCTG	Deleting the *RAD5* gene with the hphMX4 cassette
RAD5/test SP	CATTATTCCTTACTGCTAAGCGCAT	Verification of the *RAD5* deletion, used in pair with KanH-R
RAD5/test ASP	TTCTCTCGTCTTCGTCTGTGTCCAT	Verification of the *RAD5* deletion, used in pair with KanT-F
TOF1/SP	GTAAGTCGCCTCACATATGATAATACCATCTAGCTTGTGGGGTTTAGTGTATCTTCAGCTGAAGCTTCGTACGC	Deleting the *TOF1* gene with the hphMX4 cassette
TOF1/ASP	TCTGTAGCTCTTATGCTTTCAATACTTGGTATGGATCCACCAAACAAGCTCGTATCATAGGCCACTAGTGGATCTG	Deleting the *TOF1* gene with the hphMX4 cassette
TOF1/test SP	GTATGAATTGCTTCCCTGTGGAAA	Verification of the *TOF1* deletion, used in pair with KanH-R
TOF1/test ASP	GACATCGTCATCAGAGAGTTGATT	Verification of the *TOF1* deletion, used in pair with KanT-F
KR52t-F	CGAATGGCGTTTTTAAGCTATTTTGCCACTGAGAATCAACAAATGCAAACAAGGAGGTTGCCAGATCTGTTTAGCTTGCCT	Deleting the *RAD52* gene with the hphMX4 cassette
KR52h-R	GGTTTCACGCGGTACTTGATTCCCAGCCCCTTCTAGCATATGAGGCCCCAGTTCTTTATCATCGATGAATTCGAGCTCGTT	Deleting the *RAD52* gene with the hphMX4 cassette
E52st-F	GAAGCGTTTCAAGTAGGCTTG	Verification of the *RAD52* deletion, used in pair with KanT-F
E52M-R	CGTCTTCTCAATCACACTTACTC	Verification of the *RAD52* deletion, used in pair with KanH-R
KanH-R	TGGGGATGTATGGGCTAAATGTA	Verification of the hphMX4 cassette integration, reverse primer

KanT-F	CGTCAATCGTATGTGAATGCTG	Verification of the hphMX4 cassette integration, forward primer
Hyg_sp_F	CACTCGTCCGAGGGCAAAGGAAT	Verification of the hphMX4 cassette integration, forward primer
Hyg_sp_R	CGTCGCGGTGAGTTCAGGCTTT	Verification of the hphMX4 cassette integration, reverse primer
Nat_sp_F	GAGCAGGCGCTCTACATGAGCAT	Verification of the natMX6 cassette integration, forward primer
Nat_int_chk_R	GTAAGCCGTGTCGTCAAGAGTGGT	Verification of the natMX6 cassette integration, reverse primer
HIS3H-R	CCTGTGTGGACGTTAATCACTTGCGAT	Verification of the *HIS3* cassette integration, reverse primer
HIS3T-F	CAGGCCGTACGCAGTTGTCGAACT	Verification of the *HIS3* cassette integration, forward primer
MRC1DF HIS3	CTAATTATCAAAGCTATCTTGTCCGCTTTCAAAAAGTTTATTCGATTTTGGGTGTCGGGGCTGGCTTAA	Deleting the *MRC1* gene with the *HIS3* cassette
MRC1DR HIS3	GACAAACAACTAAGGAAGTTCGTTATTCGCTTTTGAACTTATCACCAAATCTCCTTACGCATCTGTGCGGTA	Deleting the *MRC1* gene with the *HIS3* cassette
MRC1TR	CTCTACTGGCTCTCATATGCAGA	Verification of the *MRC1* deletion, used in pair with HIS3T-F
MRC1TF	GCAAGATGCTTTGAATACAGAACT	Verification of the *MRC1* deletion, used in pair with HIS3H-R
TBF1-wt-CMyc-F	TGGCAATAGTACATCGGACAATACAG	Amplification of the *TBF1-13xMyc-HIS3MX6* allele for transformation, forward primer
TBF1-di-CMyc-F	AACCGCTTCAATGACATCATCCAAAG	Amplification of the *tbf1Δi-13xMyc-HIS3MX6* allele for transformation, forward primer
TBF1-CMyc-R	CAGGTTGCTAGAAGATAGGGTGAC	Amplification of the *TBF1-13xMyc-HIS3MX6*/*tbf1Δi-13xMyc-HIS3MX6* alleles for transformation, reverse primer
TBF1_di-chk_F	GCTACTAGACTTAATCTTCCCATCCAA	Checking the deletion of I-domain

TBF1_di_chk-R	TCAGTTGTACCTGCGTCCTGTTCTTC	Checking the deletion of I-domain
TBF1_CMyc_3chk-R	GAACGTGACGAATAATGTTGAAGAGG	Checking the integration of the *TBF1-13xMyc-HIS3MX6*/*tbf1Δi-13xMyc-HIS3MX6* alleles
HIS3MX6_Sp-F	CTATACTGCTGTCGATTCGATACTAAC	Checking the integration of the *TBF1-13xMyc-HIS3MX6*/*tbf1Δi-13xMyc-HIS3MX6* alleles
ADH1_t_F	TTCTTGAGTAACTCTTTCCTGTAGG	Checking the integration of the *TBF1-13xMyc-HIS3MX6*/*tbf1Δi-13xMyc-HIS3MX6* alleles
5'UAS_TBF1-F	CATTTGAAGCCCTAATATTCTGCAC	Checking the integration of the *TBF1-13xMyc-HIS3MX6*/*tbf1Δi-13xMyc-HIS3MX6* alleles
ChrV-fwd	GGCTGTGGTTTCAGGGTCCATAAAGC	Amplification of the chromosome V-specific product (Reference), forward
ChrV-rev	CTGGGCAATTTCATGTTTCTTCAACACC	Amplification of the chromosome V-specific product (Reference), reverse
VID22DTF	TATGGAAGATACTGACTTGCAGTTTGTAAAGTGTCCAAGTCGTAATCCACCAGCTGAAGCTTCGTACGC	Deleting the *VID22* gene with the *natMX6* or *hphMX4* cassette
VID22DRC	GGAAAGAGAGAGTTTGCTGGCAGCATCCACAGGATAAACAAGCACGAGAGCATAGGCCACTAGTGGATCTG	Deleting the *VID22* gene with the *natMX6* or *hphMX4* cassette
ENV11DTF	AGATTAAACTGTGAATTCAAAAGTTTGATTTTAATTGTTTCCTCACTCAACAGCTGAAGCTTCGTACGC	Deleting the *ENV11* gene with the *hphMX4* cassette
ENV11DRC	GTCTTCCGAAATAGCATATGTACCAATCTACTCAATCATTAATATGAACACATAGGCCACTAGTGGATCTG	Deleting the *ENV11* gene with the *hphMX4* cassette
VID22TF	CCTCTAACACCAAGGTCTCTTCCA	Verification of the *VID22* deletion, used in pair with Nat_sp_R or Hyg_sp_R
VID22TR	CCGTTGCTTTGTTCCAGATGGTA	Verification of the *VID22* deletion, used in pair with Nat_sp_F or Hyg_sp_F
VID22_in_F	AACTTGCCTCTGTCGTTCGTAG	Verification of the *VID22* deletion, used in pair with VID22_chk_in_R

VID22_in_R	TTCGGGAAAAAGGGTTGGCTATC	Verification of the *VID22* deletion, used in pair with VID22_chk_in_F
ENV11TF	CCGCTGTGTACTTGGAAACAAGA	Verification of the *ENV11* deletion, used in pair with Hyg_sp_R
ENV11TR	GCAGAAATGTAAGTCCCTCAGTAGGT	Verification of the *ENV11* deletion, used in pair with Hyg_sp_F
ENV11_in_F	TTTCCATAGCGGGAGTGTGAGG	Verification of the *ENV11* deletion, used in pair with ENV11_chk_in_R
ENV11_in_R	CAATGGAGCGGAAGAAGCAAAG	Verification of the *ENV11* deletion, used in pair with ENV11_chk_in_F
VID22_Myc_HIS3MX6_F	CAATTCGTGGCAAGTAAAGTGGATTACGACTTGGACACTTTACAAACTGCAAGTCAGTATCTTCCACGGATCCCCGGGTTAATTAACG	Amplification of the *VID22-13xMyc-HIS3MX6* allele for transformation, forward primer
VID22_Myc_HIS3MX6_R	CCCATTTTGATTAATCATTTCCTTAGCTGCCCAAAGAATTGCACGCTTACAGTGAGGAGTAGTGATACATGAATTCGAGCTCGTTTAAACTG	Amplification of the *VID22-13xMyc-HIS3MX6* allele for transformation, reverse primer
HIS3MX6_chk_F	ACGTTCCCTCAACCAAAGGT	Checking the integration of the *VID22-13xMyc-HIS3MX6* alleles. Used in combination with oligo VID22TF
VID22_chk_F	CCCAGAGTACATCCCGATTT	Checking the integration of the *VID22-13xMyc-HIS3MX6* alleles. Used in combination with oligo KanH-R
TBF1_MYCdel_PRCC_F	GAAGCAATGGGTGCTGTCCGGTTTTAGAGCTAGAAATAGCAAGTTAAAATAAGG	Amplification of the pRCC-N plasmid to delete the 13xMyc tag from the *tbf1Δi-13xMyc-HIS3MX6* allele, forward primer
TBF1_MYCdel_PRCC_R	CGGACAGCACCCATTGCTTCCGATCATTTATCTTTCACTGCGGAG	Amplification of the pRCC-N plasmid to delete the 13xMyc tag from the *tbf1Δi-13xMyc-HIS3MX6* allele, reverse primer
TBF1_MYCdel_template	GTACATCGGACAATACAGGTTTTGATCCTCATTTAGAAGATGGGATGTAGCGCGCTTAAATGAACAGACTTCTAATTTCTATTGAACCACTCACTATTA	Used as a repair template to delete the 13xMyc tag from the *tbf1Δi-13xMyc-HIS3MX6* allele

HIS3MX6_chk_R	ACCTTTGGTTGAGGGAACGT	Checking the integration of the *VID22-13xMyc-HIS3MX6* alleles. Used in combination with oligo VID22_chk_F
HIS3MX6_chk_F2	GCTATTTCGCTGAATGGTGG	Checking the integration of the *VID22-13xMyc-HIS3MX6* alleles. Used in combination with oligo VID22TF
HIS3MX6_chk_R2	CCACCATTCAGCGAAATAGC	Checking the integration of the *VID22-13xMyc-HIS3MX6* alleles. Used in combination with oligo VID22_chk_F
pAMP	GGCGGAACCCCTATTTGTTT	Amplification of the probe for 2D gels
AmpR	TGAGAATAGTGTATGCGGCG	Amplification of the probe for 2D gels
UIRL1-BsrGI	GCATATGTACAGAAGTAACAAAGGAACCTAGAGGGTA	Amplification of the $(CCCTAA)_{60}$ repeat or non-repetitive control to integrate into the pRS425 plasmid
UIRL2-BsrGI	GCAGTTGTACATACAGATCAGTCAATATAGGAGGTT	Amplification of the $(CCCTAA)_{60}$ repeat or non-repetitive control to integrate into the pRS425 plasmid

Table S3.
Rates of 5-FOA^R clones and Htel expansions in strains with various repeat lengths in both orientations, (η x 10^{-6}).

	**$(TTAGGG)_{25}$**	**$(TTAGGG)_{32}$**	**$(TTAGGG)_{39}$**	**$(TTAGGG)_{60}$**
5-FOA^R	1.14(0.75-1.59)	3.23(2.2-4.4)	20.7 (13.8-28.6)	1210 (912-1550)
Expansions	0.2 (0.06-0.4)	0.5 (0.2-1.0)	5.41(1.6 -10.7)	399 (173 -691)
	**$(CCCTAA)_{25}$**	**$(CCCTAA)_{32}$**	**$(CCCTAA)_{38}$**	**$(CCCTAA)_{60}$**
5-FOA^R	1.94 (1.56-2.35)	24.4 (18.1 -31.5)	452 (325-594)	NA
Expansions	0.32 (0.15-0.55)	12.1 (4.92 -21.4)	569 (324-861)	NA

95% Confidence limits are in the parentheses. 5-FOA^R and expansion rates were calculated by VZ-MLE as described earlier (8).

Table S4.
Ratio of mutations and other events among sequenced 5-FOA^R clones in strains with different orientation of the Htel repeat.

	$(TTAGGG)_{25}$	$(CCCTAA)_{25}$	$(TTAGGG)_{39}$	$(CCCTAA)_{38}$
# analyzed colonies	44	48	48	48
Mutations	29.5%	37.5%	0%	0%
Big Deletions or GCRs*	22.7%	6.25%	0%	0%

*Deletions are confirmed by sequencing of the *URA3* gene; potential gross chromosomal rearrangements involving the *URA3* gene denote the colonies where we failed to get the PCR product with primers flanking the Htel tract and primers flanking the entire *URA3* gene.

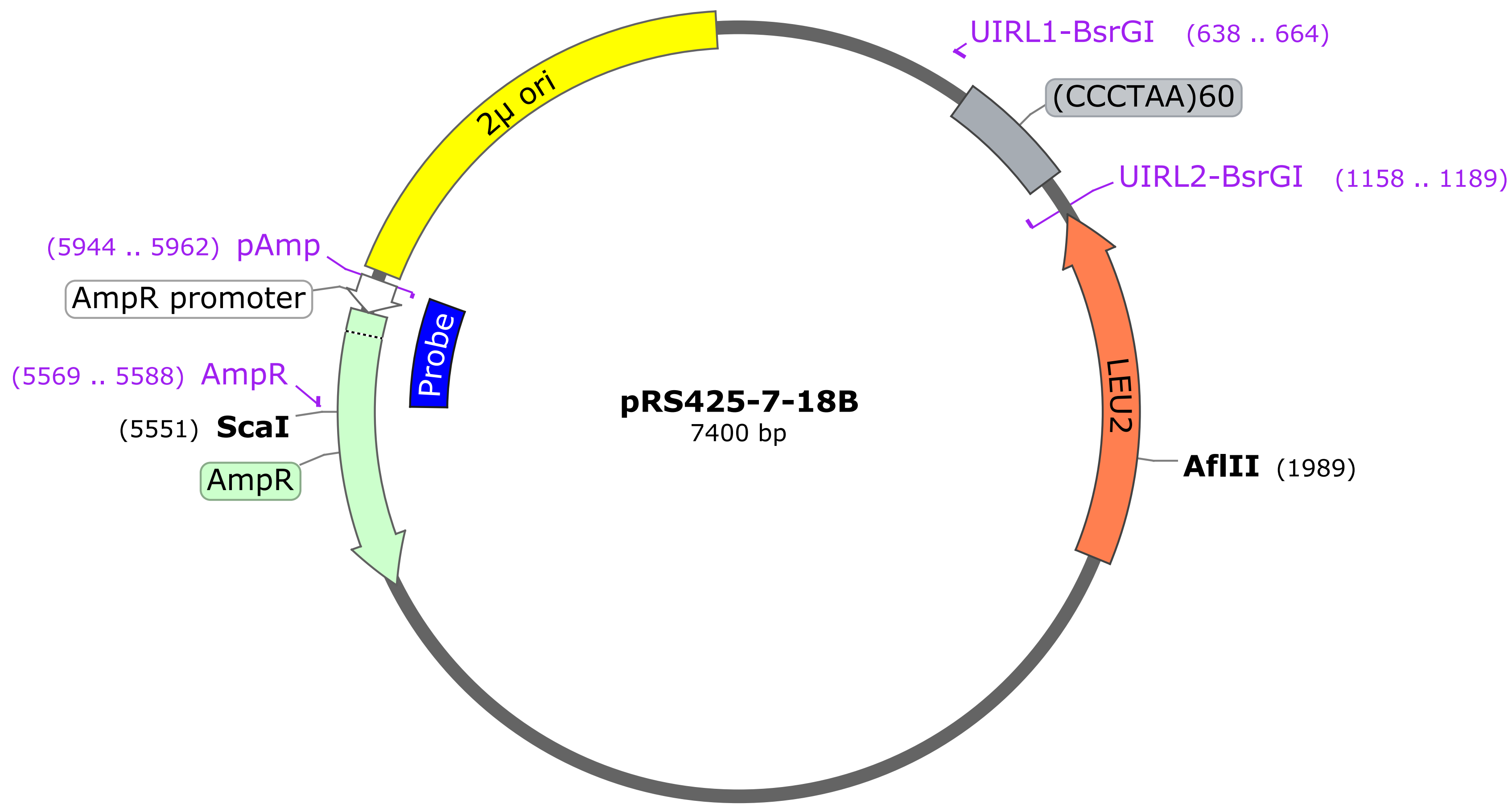

Figure S1. Schematic of the pRS425-7-18B plasmid used for 2D gel analysis. The relative positions of the most relevant features are indicated: the 2 μm origin (yellow), the bacterial ampicillin-resistance gene (AmpR) (light green), yeast auxotrophic *LEU2* marker (orange), 394 bp hybridization probe (blue), and the $(CCCTAA)_{60}$ repeat (gray). pAMP and AmpR primers were used to amplify the probe. UIRL1-BsrG1 and UIRL2-BsrGI primers were used for cloning the Htel repeats into the pRS425 plasmid. Relative positions of sites recognized by the restriction endonucleases ScaI and AflII are indicated.

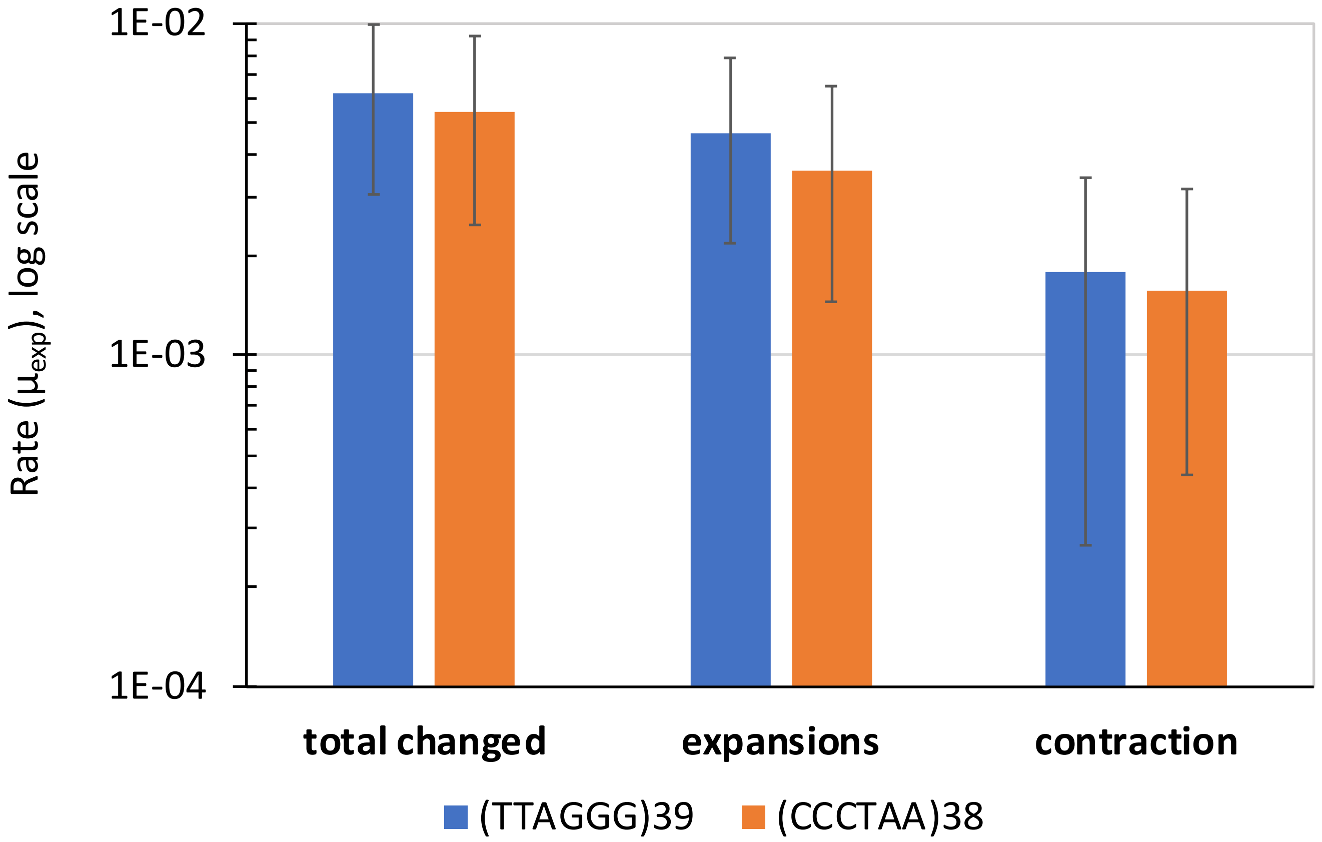

Figure S2. Rates of Htel instability (expansions and contractions) measured in non-selective conditions as described for Ytel repeats in (3). 240 clones from 12 independent cultures grown in non-selective conditions on YPD were randomly picked and analyzed by PCR and gel-electrophoresis for each tested strain.

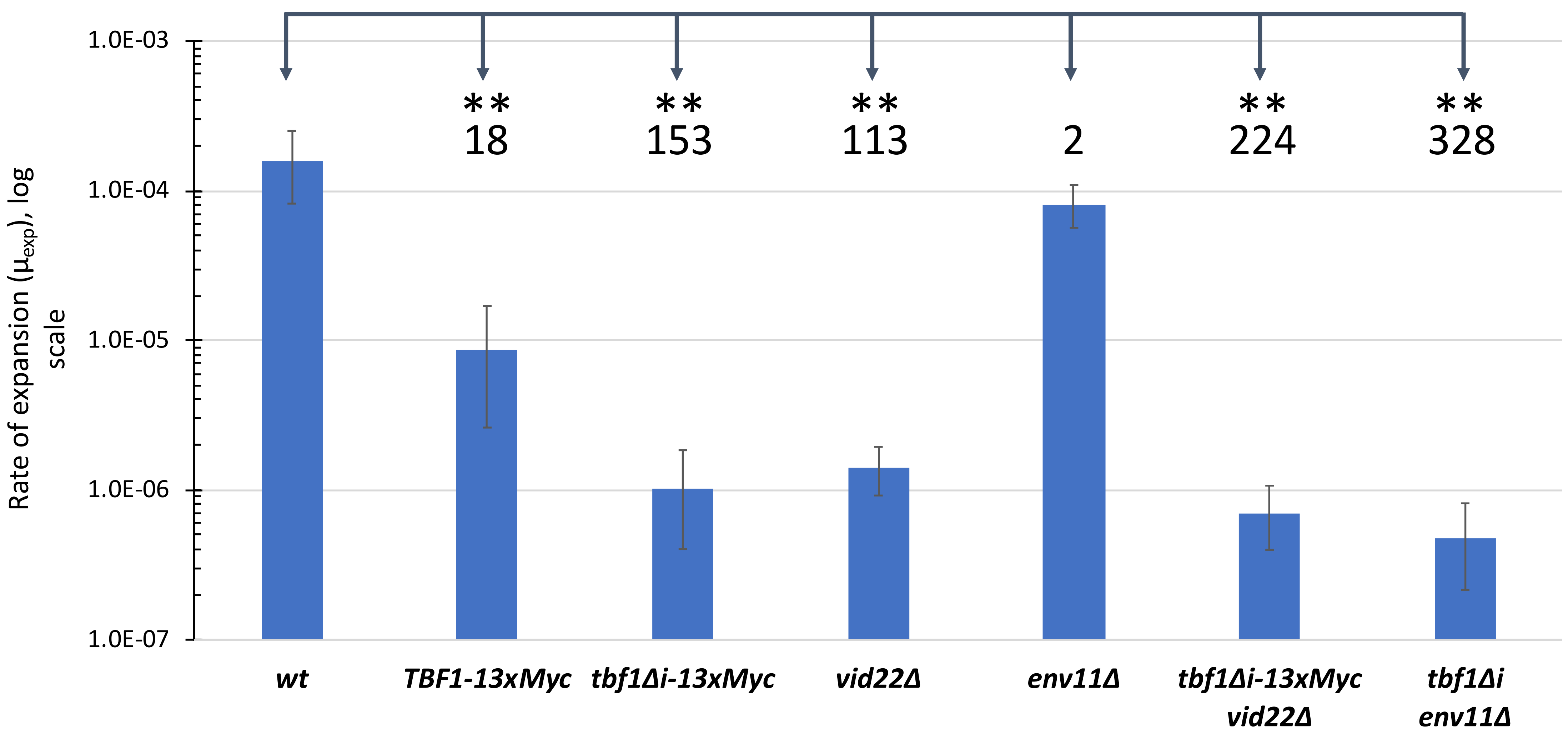

Figure S3. Expansion rate of the $(CCCTAA)_{38}$ repeat is decreased in strains carrying *TBF1-13xMyc, tbf1Δi-13xMyc* and *vid22Δ* alleles, but not in the *env11Δ* strain. Error bars represent 95% confidence intervals. Numbers above the bars indicate fold change in expansion rate in mutant strains compared to the isogenic *wt* (SMY758) strain. ** - non-overlapping 95% confidence intervals.

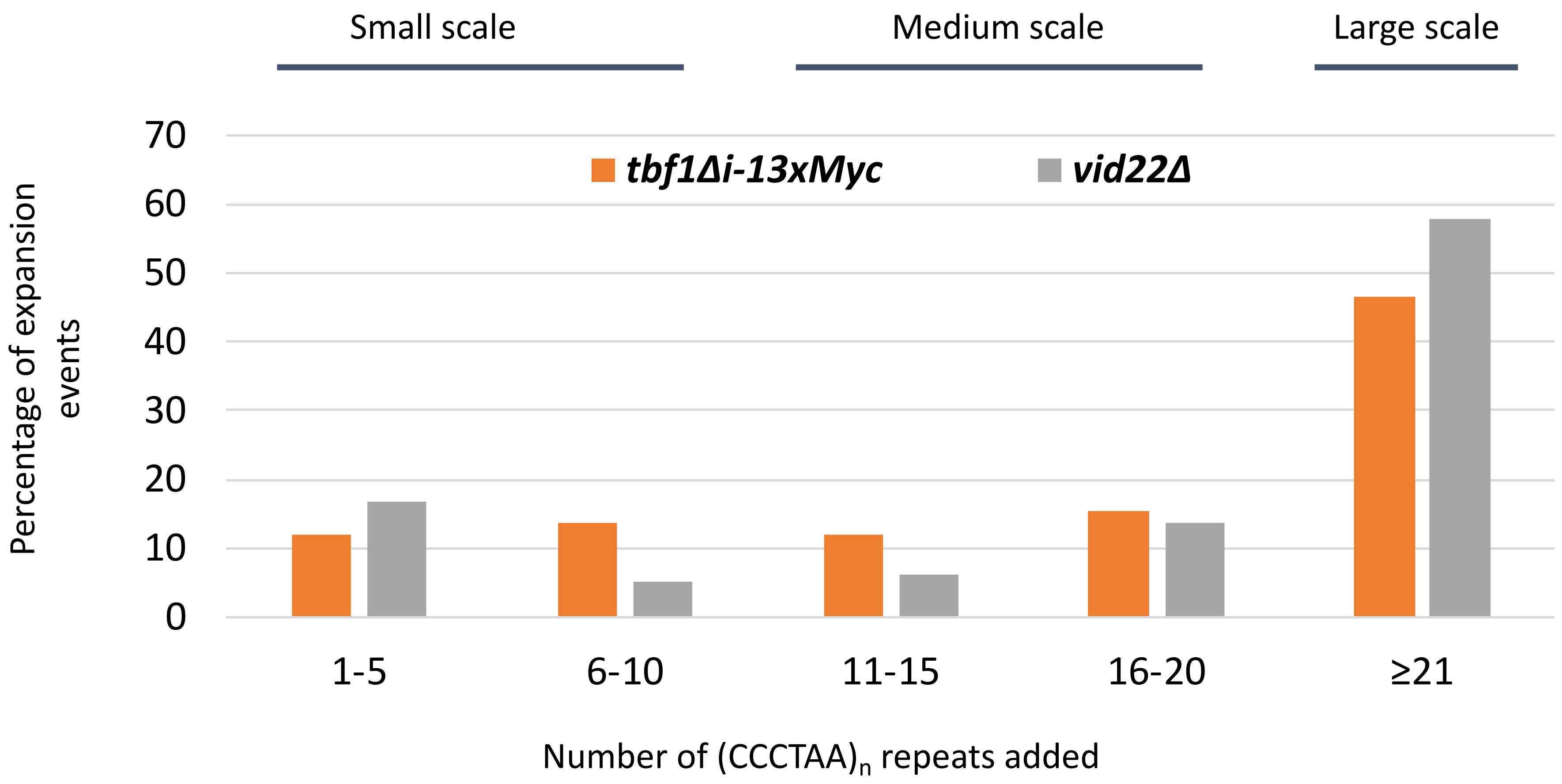

Figure S4. Length distribution of expanded repeats among 5-FOA^R clones in the *tbf1Δi-13xMyc* and *vid22Δ* strains carrying the $(CCCTAA)_{38}$ repeat.

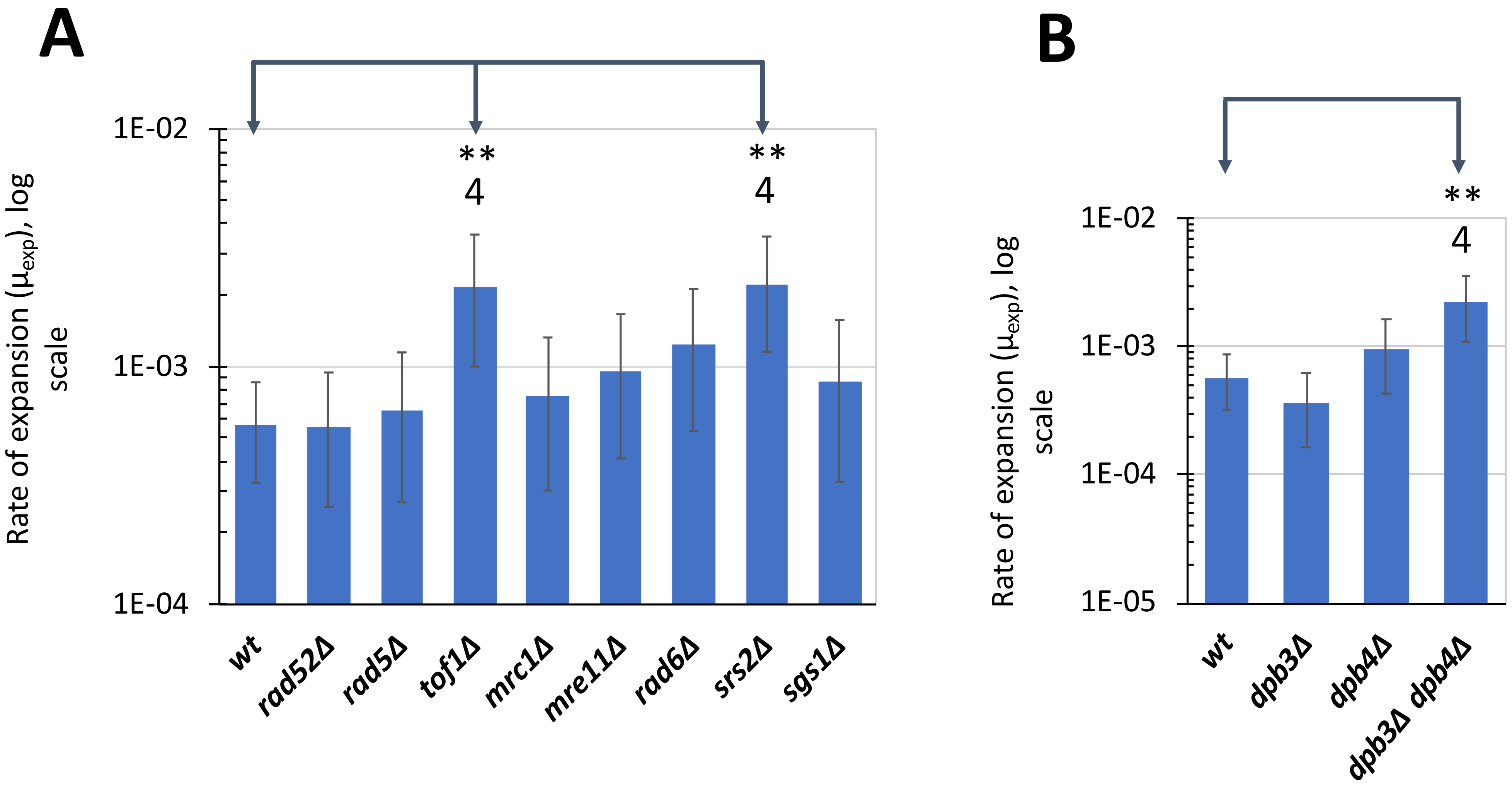

Figure S5. Genetic control of Htel repeat expansions.
A. Influence of individual knock-outs of *RAD52, RAD5, TOF1, MRC1, MRE11, RAD6, SRS2* and *SGS1* genes on the expansion rate of $(CCCTAA)_{38}$ repeat. Numbers above the bars indicate fold change in expansion rate in *tof1* and *srs2Δ* strains compared to the isogenic *wt* (SMY758) strain. ** - non-overlapping 95% confidence intervals compared to *wt*. **B.** Influence of accessory subunits of the DNA polymerase epsilon on the expansion rate of the $(CCCTAA)_{38}$ repeat. Shown are individual and double knockouts of genes encoding the Dpb3 and Dpb4 subunits of Pol ε. Numbers above the bars indicates fold change in expansion rate compared to the isogenic *wt* (SMY758) strain. ** - non-overlapping 95% confidence intervals compared to *wt*.
